# Molecular insights into LINC complex architecture through the crystal structure of a luminal trimeric coiled-coil domain of SUN1

**DOI:** 10.3389/fcell.2023.1144277

**Published:** 2023-06-21

**Authors:** Manickam Gurusaran, Jelle J. Biemans, Christopher W. Wood, Owen R. Davies

**Affiliations:** ^1^ Wellcome Centre for Cell Biology, Institute of Cell Biology, University of Edinburgh, Edinburgh, Scotland, United Kingdom; ^2^ Institute of Quantitative Biology, Biochemistry and Biotechnology, University of Edinburgh, Edinburgh, Scotland, United Kingdom

**Keywords:** LINC complex, nuclear envelope, SUN1, KASH5, X-ray crystallography, molecular dynamics, biophysics

## Abstract

The LINC complex, consisting of interacting SUN and KASH proteins, mechanically couples nuclear contents to the cytoskeleton. In meiosis, the LINC complex transmits microtubule-generated forces to chromosome ends, driving the rapid chromosome movements that are necessary for synapsis and crossing over. In somatic cells, it defines nuclear shape and positioning, and has a number of specialised roles, including hearing. Here, we report the X-ray crystal structure of a coiled-coiled domain of SUN1’s luminal region, providing an architectural foundation for how SUN1 traverses the nuclear lumen, from the inner nuclear membrane to its interaction with KASH proteins at the outer nuclear membrane. In combination with light and X-ray scattering, molecular dynamics and structure-directed modelling, we present a model of SUN1’s entire luminal region. This model highlights inherent flexibility between structured domains, and raises the possibility that domain-swap interactions may establish a LINC complex network for the coordinated transmission of cytoskeletal forces.

## Introduction

**FIGURE 1:**
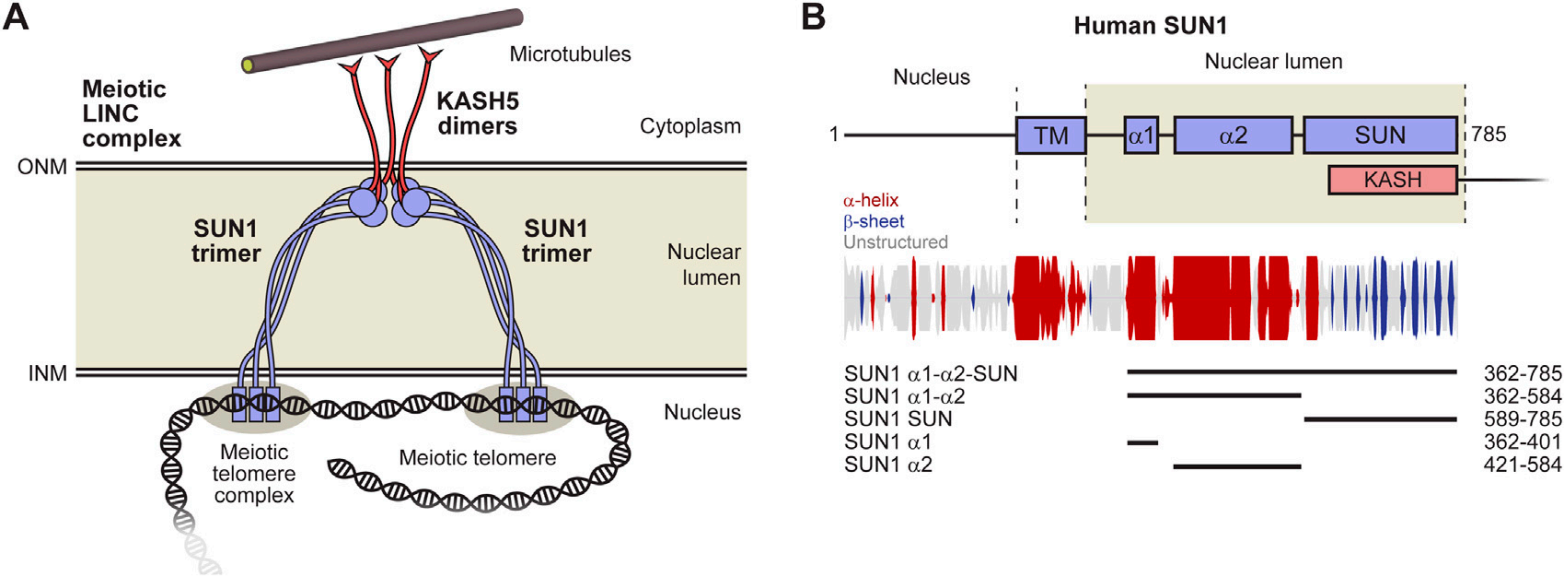
The LINC complex. **(A)** The LINC complex consists of interacting SUN and KASH proteins that bind to nuclear and cytoskeletal components, respectively. The luminal region of SUN proteins is thought to consist of a trimeric coiled-coil that bridges between the inner (INM) and outer (ONM) nuclear membranes. SUN trimers terminate in globular domains that interact head-to-head in 6:6 complexes with KASH proteins immediately below the ONM. Whilst we have depicted the LINC complex with a 6:6 stoichiometry, owing to its observation in crystallographic and biochemical studies (Sosa et al., 2012; Wang et al., 2012; Zhou et al., 2012; Cruz et al., 2020; Gurusaran and Davies, 2021), other stoichiometries have been proposed to form in proximity of the ONM *in vivo* (Jahed et al., 2021). Additional components, such as dynein and dynactin, are not depicted to enhance clarity. **(B)** Schematic of the human SUN1 sequence in which its transmembrane (TM) and luminal regions are highlighted. Secondary structure prediction is shown with propensity indicated by peak height (α-helix, red; β-sheet, blue; unstructured, grey) (Drozdetskiy et al., 2015). The principal constructs used in this study are indicated as luminal coiled-coil domains α1 and α2, along with the C-terminal KASH-interacting SUN domain.

The Linker of Nucleoskeleton and Cytoskeleton (LINC) complex traverses both inner and outer nuclear membranes to provide physical connections between the cytoskeleton and nuclear contents (Starr and Fridolfsson, 2010) (Figure 1A). The central role of the LINC complex in force transduction is exemplified by its essential function in meiosis. During the first meiotic division, the telomeric ends of chromosomes become tethered to the inner nuclear membrane by the meiotic telomere complex (Shibuya et al., 2015; Dunce et al., 2018b). Here, they bind to the meiotic LINC complex, which transmits microtubule-generated forces to chromosome ends by acting as a transmembrane dynein activating adapter (Horn et al., 2013b; Spindler et al., 2019; Agrawal et al., 2022; Garner et al., 2022). This results in rapid chromosome movements that are thought to facilitate recombination searches and the establishment of homologous chromosome pairs that are necessary for reductive division and crossing over (Shibuya et al., 2014; Lee et al., 2015). Hence, the meiotic LINC complex is required for fertility (Horn et al., 2013b). In addition to meiosis, and other specialised roles such as in sound perception in the inner ear (Horn et al., 2013a), the LINC complex has generalised functions in determining nuclear structure, shape and position by transmitting active and reactive tension forces to the nuclear lamina and chromatin (Crisp et al., 2006; Luxton et al., 2010; Alam et al., 2015). Hence, the LINC complex is important for cellular life, and its mutations are implicated in laminopathies, including Hutchison-Gilford progeria syndrome and Emery-Dreifuss muscular dystrophy (Mejat and Misteli, 2010; Meinke et al., 2011).

The LINC complex is formed of SUN (Sad1 and UNC84 homology) and KASH (Klarsicht, ANC-1, and Syne homology) proteins (Starr and Fridolfsson, 2010; Meinke and Schirmer, 2015). The SUN protein has an N-terminal nuclear region, crosses the inner nuclear membrane, and then traverses the nuclear lumen (perinuclear space) to position its C-terminal SUN domain immediately below the outer nuclear membrane (Figures 1A, B). Here, it interacts with the eponymous KASH domain, located at the C-terminus of the KASH protein, which then crosses the outer nuclear envelope, and has a large cytoplasmic domain that mediates interactions with the cytoskeleton (Sosa et al., 2012; Wang et al., 2012; Zhou et al., 2012).

The LINC complex is formed by a family of SUN and KASH proteins, which have both generalised and tissue-specific functions. In mammals, there are five SUN proteins (SUN1-5), of which SUN1 and SUN2 are generally expressed and exhibit partial redundancy in nuclear anchorage (Lei et al., 2009; Zhang et al., 2009). SUN1 is essential for meiosis as its disruption in mice leads infertility owing to failure of chromosome synapsis (Ding et al., 2007). Hence, whilst SUN2 is expressed in meiosis and contributes to meiotic telomere attachment (Schmitt et al., 2007; Link et al., 2014), it does not provide redundancy for the meiotic function of SUN1. The remaining SUN proteins, SUN3-5, are specifically expressed in the later stages of spermatogenesis, where they each perform essential roles in sperm head formation (Pasch et al., 2015; Gao et al., 2020; Zhang et al., 2021). There are six mammalian KASH proteins, of which four are Nesprins (Nuclear Envelope Spectrin Repeat proteins). Nesprin-1 and Nesprin-2 are generally expressed, and perform overlapping roles in nuclear anchorage through interactions between their cytoplasmic spectrin-repeat domains and actin (Banerjee et al., 2014; Sakamoto et al., 2017; Zhou et al., 2018). Nesprin-3 is widely expressed are maintains nuclear integrity by interacting with intermediate filaments and microtubules via plectin, BPAG1 and MACF (Wilhelmsen et al., 2005; Ketema and Sonnenberg, 2011). Nesprin-4 is also widely expressed, and functions in microtubule-dependent nuclear positioning by binding to the motor protein kinesin-1 (Roux et al., 2009). It also has an essential role in hearing through a specific function in the outer hair cells of the inner ear (Horn et al., 2013a). KASH5 is a meiosis-specific coiled-coil protein that functions as a dynein activating adapter that transmits microtubule forces to meiotic chromosome and is essential for their synapsis and fertility (Horn et al., 2013b; Agrawal et al., 2022; Garner et al., 2022). The final KASH protein, JAW1/LRMP/IRAG2, interacts with microtubules, and is required to maintain nuclear shape and Golgi structure (Kozono et al., 2018; Okumura et al., 2023). Hence, the combination of five SUN proteins (and their multiple isoforms) and six KASH proteins achieve the widespread, varied and essential functions of the LINC complex in mammals. Owing to the essential roles of SUN1 and KASH5 in meiotic chromosome synapsis and fertility, this study focusses on the meiotic SUN1-KASH5 LINC complex.

The mechanism of force transduction by the LINC complex is inherently defined by its molecular architecture. Structural work has mostly focussed on the SUN-KASH domain interaction that binds together LINC components (Sosa et al., 2012; Wang et al., 2012; Zhou et al., 2012; Cruz et al., 2020; Gurusaran and Davies, 2021). The SUN domain is a globular structure, which upon interaction with KASH domains, forms a trimer preceded by a short coiled-coil (Sosa et al., 2012; Wang et al., 2012; Zhou et al., 2012). Nesprin-1/2/3, Nesprin-4 and KASH5 interact with the SUN domain through a common C-terminal motif and diverse N-terminal interfaces (Cruz et al., 2020; Gurusaran and Davies, 2021). SUN1-KASH complexes are 6:6 structures, formed of two SUN domain trimers associated head-to-head through KASH-mediated interactions, whilst SUN2-KASH complexes form 6:6 and higher order assemblies (Gurusaran and Davies, 2021). Whilst other SUN-KASH complex stoichiometries have been proposed to form *in vivo*, particularly in proximity to the outer nuclear membrane (Jahed et al., 2021), the 6:6 complex is the only structure that has hitherto been observed in crystal structures and in solution (Sosa et al., 2012; Wang et al., 2012; Zhou et al., 2012; Cruz et al., 2020; Gurusaran and Davies, 2021). In absence of KASH-binding, isolated SUN domains remain monomeric, held in autoinhibited complexes by preceding α-helices that otherwise form the SUN-KASH complex coiled-coils (Nie et al., 2016; Jahed et al., 2018a; Jahed et al., 2018b; Xu et al., 2018). There are no structures of cytoplasmic regions of KASH proteins other than short stretches of Nesprin-1/2 (Lim et al., 2021), although we know that KASH5 is a dimer (Agrawal et al., 2022; Garner et al., 2022). There is also a structure of a short nuclear region of SUN1 in a meiotic regulatory complex with SpeedyA-CDK2 (Chen et al., 2021).

The luminal region of SUN proteins upstream of the SUN domain is thought to consist of a long trimeric coiled-coil that passes between nuclear membranes (Jahed et al., 2021). This is based on the short trimeric coiled-coils that emanate from each SUN trimer of the SUN-KASH complex (Sosa et al., 2012; Wang et al., 2012; Zhou et al., 2012), gel filtration and biophysical studies of SUN2 (Jahed et al., 2018b), and the crystal structure of a luminal CC1 trimeric coiled-coil of SUN2 (Nie et al., 2016). The alteration of oligomer state along the LINC complex axis, between SUN trimer, SUN-KASH 6:6 complex and KASH5 dimer, has been proposed to establish a branched LINC complex network suitable for cooperative force transduction (Figure 1A) (Gurusaran and Davies, 2021). Further, the geometry of SUN coiled-coil trimers emanating from SUN-KASH 6:6 complexes suggests that SUN molecules must re-orient from being perpendicular to parallel to the nuclear membrane as they pass through the nuclear lumen (Gurusaran and Davies, 2021). However, the absence of a structure of the full luminal region of a SUN protein has precluded us from visualising how this may occur at the molecular level.

Here, we report the crystal structure of a trimeric coiled-coil domain within the luminal region of SUN1, which lies upstream of a second coiled-coil trimer that corresponds to SUN2’s CC1. The two coiled-coils combine in a mutually reinforcing trimer that holds together three SUN domains for KASH-binding and induced head-to-head association. We combine our crystal structure with previous structures of SUN2 CC1 and the autoinhibited SUN domain to build a molecular model of SUN1’s entire luminal trimer, which has a length matching that of its solution structure, and which is sufficient to traverse the nuclear lumen. Further, the presence of flexible linkers between the constituent coiled-coils of SUN1 suggest that domain-swap interactions may provide additional branching for force transduction within an integrated LINC complex network.

## Results

### Crystal structure of a luminal coiled-coil domain of SUN1

**FIGURE 2:**
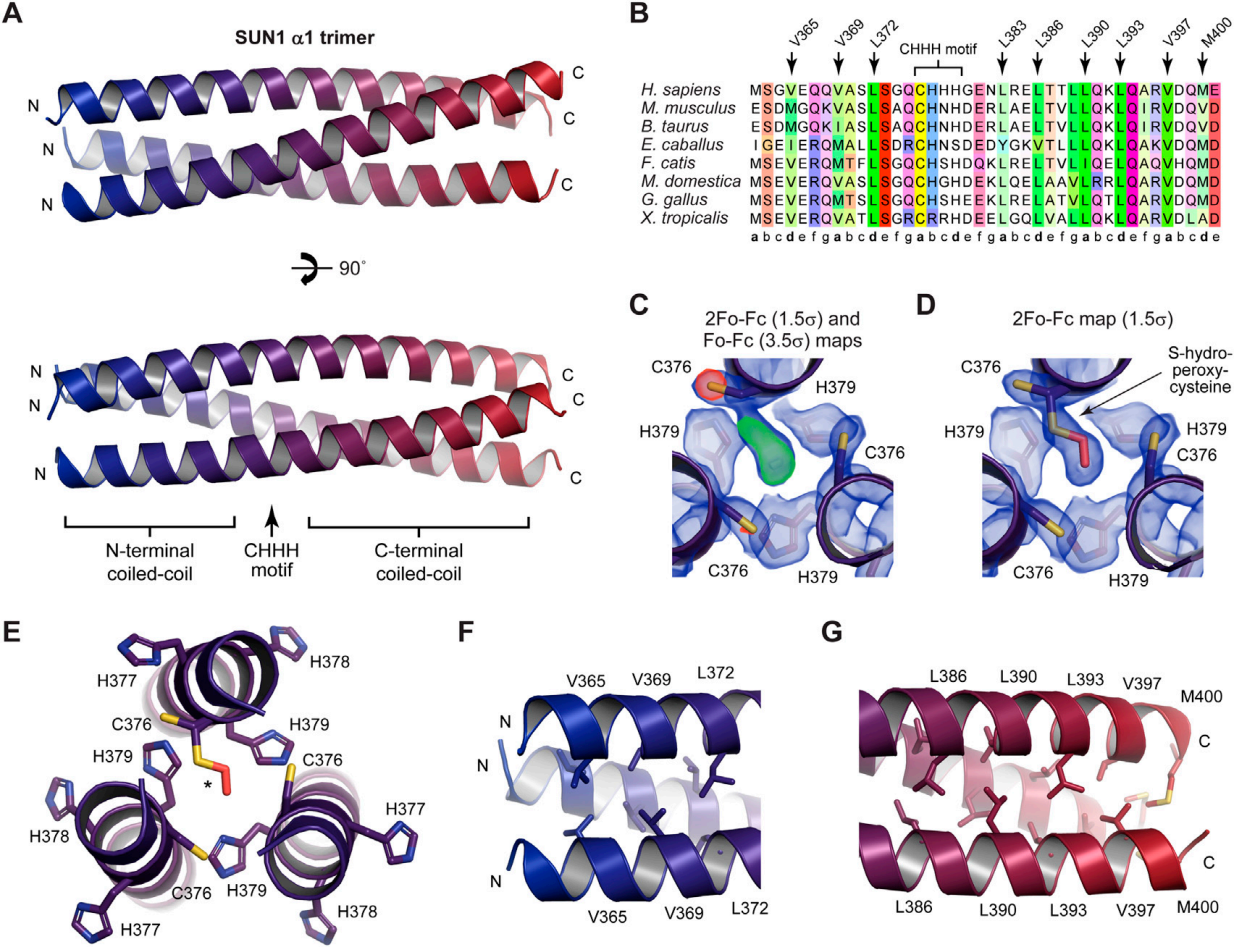
Crystal structure of the α1 luminal coiled-coil domain of SUN1. **(A)** Crystal structure of the SUN1 α1 trimeric coiled-coil. The three SUN1 chains (coloured blue to red in an N- to C-terminal direction) are arranged in a parallel configuration, in which a central CHHH motif is continuous with flanking N- and C-terminal coiled-coils. **(B)** Multiple sequence alignment of SUN1 α1, highlighting the central CHHH motif and the amino-acids at ‘a’ and ‘d’ heptad positions within the structure. Amino-acids are coloured by chemical properties and according to conservation (Waterhouse et al., 2009). **(C)** 2Fo-Fc map (blue; contoured at 1.5σ) and Fo-Fc difference map (positive difference, green; negative difference, red; contoured at 3.5σ) of the SUN1 α1 structure refined with a reduced cysteine at residue C376 of chain B. **(D)** 2Fo-Fc map (blue; contoured at 1.5σ) of the SUN1 α1 structure in which C376 of chain B was modelled with S-hydroperoxycysteine (2CO) as an alternative conformation of the reduced cysteine residue, refined with relative occupancies of 0.62 and 0.38, respectively. **(E–G)** Structural details of the **(E)** central region encompassing the 376-CHHH-379 motif **(F)** N-terminal coiled-coil formed of heptad residues V365, V369 and L372, and **(G)** C-terminal coiled-coil formed of heptad residues L386, L390, L393, V397 and M400.

The structure of SUN1’s luminal region defines how forces are transduced between the inner and outer nuclear membranes. However, we have hitherto lacked any structural information regarding the luminal region of SUN1 preceding its KASH-interacting SUN domain. On the basis of conservation and secondary structure prediction (Figure 1B), we identified a 40 amino-acid coiled-coil domain towards the beginning of human SUN1’s luminal region (amino-acids 362–401; herein referred to as α1), which was stable in solution following recombinant expression (Supplementary Figure S1). We obtained crystals of SUN1 α1 that diffracted anisotropically to a maximum resolution limit of 2.1 Å, and solved its X-ray crystal structure by molecular replacement of ideal helical fragments using *ARCIMBOLDO_LITE* in ‘coiled-coil’ mode (Caballero et al., 2018) (Table 1 and Supplementary Figure S2). This revealed a parallel trimeric coiled-coil of approximately 6 nm in length (Figure 2A), in which the “a” and “d” heptad amino-acids are conserved across vertebrates (Figure 2B). Hence, we provide the molecular structure of a trimeric coiled-coiled domain within SUN1’s luminal region.

**TABLE 1:** Data collection, phasing and refinement statistics.

	SUN1 α1
	362–401
PDB accession	8AU0
Data collection	
Space group	P2_1_
Cell dimensions	
*a*, *b*, *c* (Å)	33.31, 35.99, 46.10
α, β, γ (°)	90, 104.543, 90
Resolution (Å)	30.31—2.07 (2.11—2.07)*
*R*_meas_	0.083 (0.572)
*R*_pim_	0.026 (0.287)
*I*/σ(*I)*	8.6 (2.3)
*CC*_*1/2*_	0.997 (0.903)
Completeness (spherical) (%)	96.6 (96.4)
Redundancy	3.8 (3.9)
Refinement	
Resolution (Å)	30.31—2.07
UCLA anisotropy (Å)	2.1, 2.1, 2.4
No. reflections	5183
*R*_work_/*R*_free_	0.2438/0.2551
Cruickshank DPI (Å)	0.25
No. atoms	1,008
Protein	953
Ligand/ion	0
Water	55
*B*-factors	20.98
Protein	20.81
Ligand/ion	N/A
Water	23.84
R.m.s deviations	
Bond lengths (Å)	0.006
Bond angles (°)	0.790

^a^ Values in parentheses are for highest-resolution shell.

At the centre of the SUN1 α1 coiled-coil structure is a 376-CHHH-379 motif, in which the cysteine residues are at the “a” position of the heptad repeat (Figure 2B) and are oriented away from the coiled-coil axis (Figure 2C). We observed additional electron density for one cysteine of the trimer, which points towards the coiled-coil axis, likely representing an oxidised cysteine residue (Figure 2C). On the basis of the additional density, we modelled this as S-hydroperoxycysteine, as an alternative conformation of the reduced cysteine residue, which refined with relative occupancies of 0.62 and 0.38, respectively (Figures 2D, E). The oxidised C376 residue is packed within the core of the structure, so likely provided additional stability to the coiled-coil, and may have assisted the formation of a robust crystal system suitable for X-ray diffraction. Further, it creates an asymmetry in the structure, explaining the lack of crystallographic three-fold symmetry, with the full trimer constituting the crystal’s asymmetric unit.

The central 376-CHHH-379 motif (Figure 2E) is flanked by canonical coiled-coil interfaces. The trimeric coiled-coil on the N-terminal side is formed by heptad amino-acids V365, V369 and L372 (Figure 2F), whilst the coiled-coil on the C-terminal side is formed by heptad residues L386, L390, L393, V397 and M400 (Figure 2G). Overall, the structure can be considered as a single trimeric coiled-coil in which the heptad patterns of N-terminal and C-terminal coiled-coil regions are continuous through the intervening “CHHH”-motif region (Figure 2B).

### The SUN1 α1 structure is stable during molecular dynamics simulations

**FIGURE 3:**
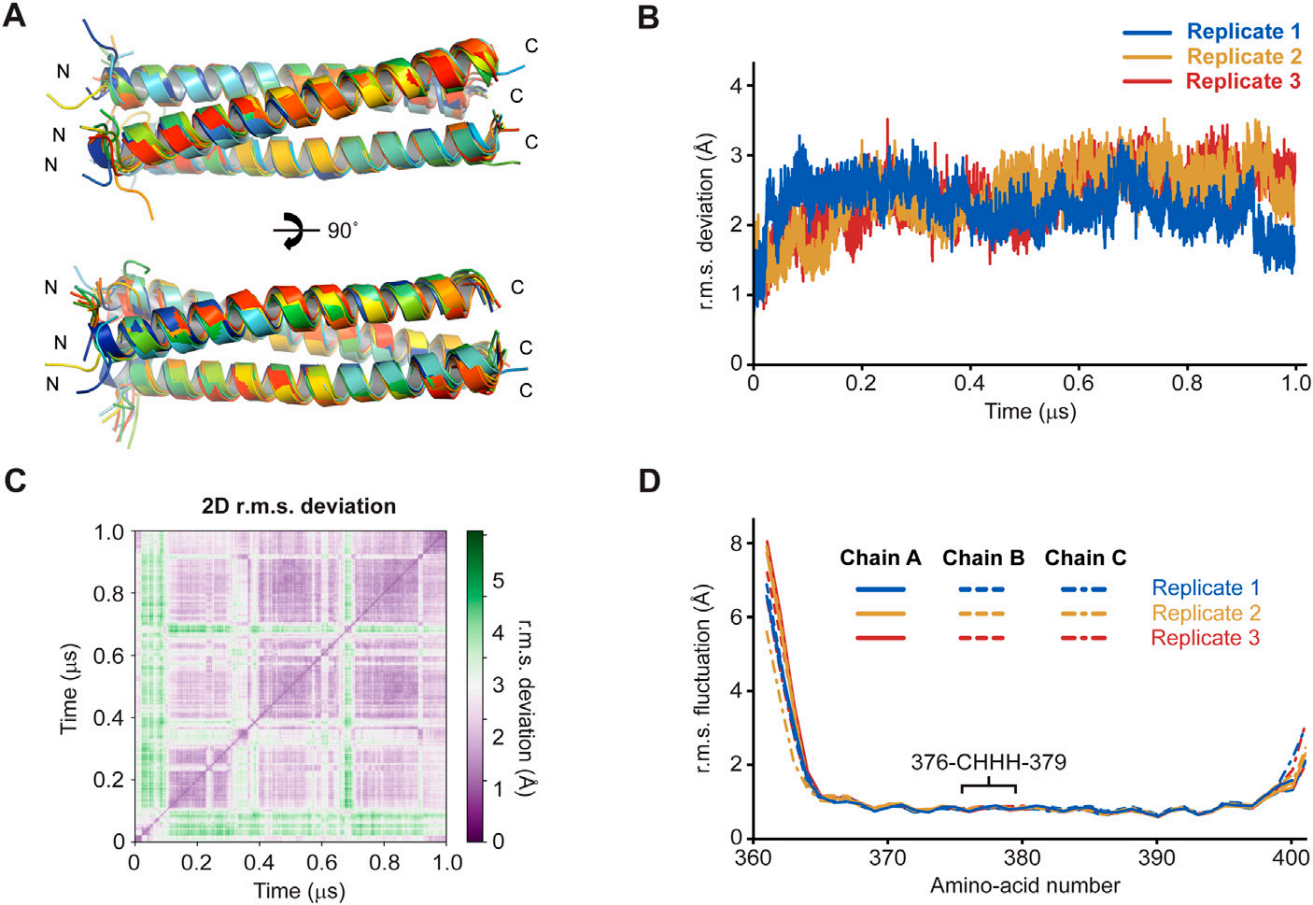
Molecular dynamics simulations of the SUN1 α1 structure over 1-μs trajectories at 37°C (n = 3). **(A)** Superimposed SUN1 α1 trimeric coiled-coil structures at 100-ns intervals of a representative trajectory, coloured from blue (0 ns) to red (1 µs). **(B)** Overall r.m.s deviations and **(C)** 2D r.m.s deviations (corresponding to panel A) across 1-μs trajectories (2D r.m.s deviations for the remaining replicates are shown in Supplementary Figure S4). **(D)** Individual amino-acid r.m.s fluctuations following 1-μs trajectories, shown for all chains of the trimer (solid, dashed and dashed/dotted), and indicating the position of the central CHHH motif.

We assessed whether the SUN1 α1 trimeric coiled-coil is stable, or could form alternative conformations, through molecular dynamics simulations. The structure was modified to remove the S-hydroperoxycysteine conformation, leaving only reduced C376 residues with full occupancy, and was subjected to molecular dynamics simulations at 37°C. In three replicates of 1-µs simulations in explicit solvent, the structure remained intact and retained its hydrophobic core (Figure 3A). The overall r.m.s deviation was constant throughout the runs, at values of typically between 1.5–3 Å (Figures 3B, C and Supplementary Figure S3. Further, local r.m.s fluctuations were below 1 Å for the coiled-coil α-helices of all chains between amino-acids 365–397, including the central 376-CHHH-379 motif (Figure 3D). The local r.m.s fluctuations had higher values at the N- and C-termini, consistent with splaying apart of helices at the end of the coiled-coil. Notably, these values were greater at N-termini (up to 8 Å) than C-termini (up to 3 Å), consistent with the more extensive coiled-coil on the C-terminal side of the “CHHH”-motif providing greater stability (Figure 3D). Further, α-helical secondary structure was retained throughout the simulations (Supplementary Figure S3). These findings are consistent with the SUN1 α1 crystal structure representing its principle trimeric conformation. Further, as the model contained only reduced C376 amino-acids, these molecular dynamics simulations are consistent with oxidation having occurred as an artefact of crystallisation rather than being a necessary requirement for complex formation.

### The SUN1 α1 trimer is stabilised by zinc coordination

**FIGURE 4:**
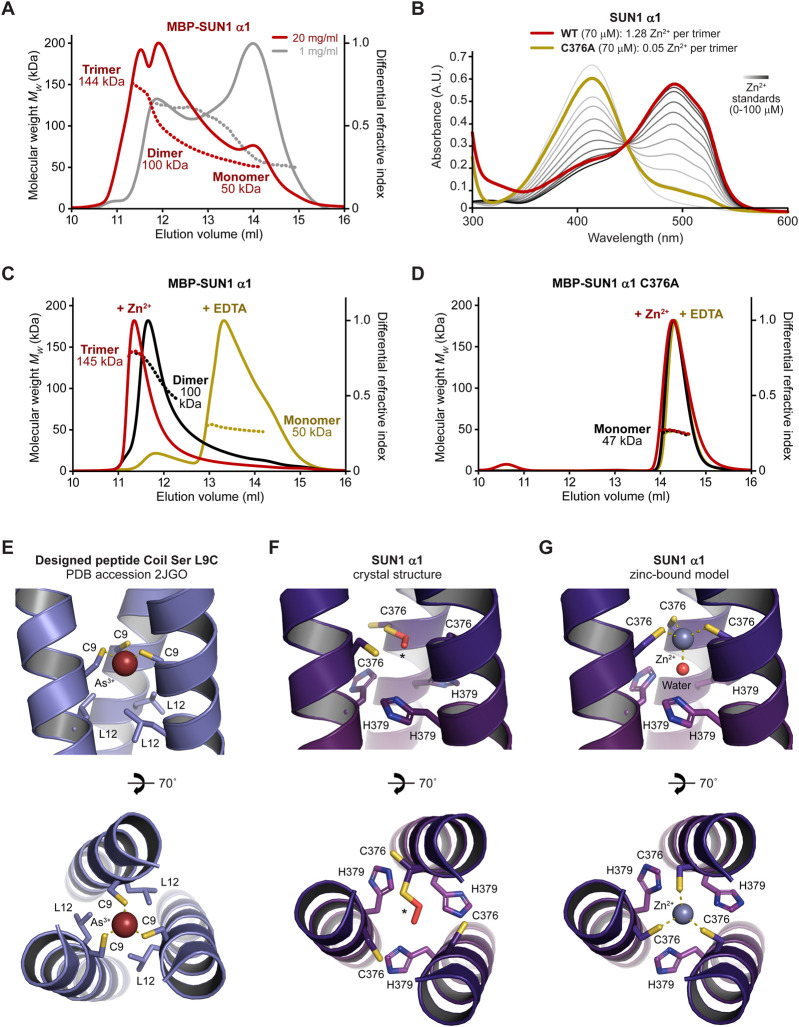
The SUN1 α1 trimer is stabilised by zinc coordination. **(A)** SEC-MALS analysis in which differential refractive index (dRI; solid lines) is shown with fitted molecular weights (*Mw*; dashed lines) plotted across elution peaks. MBP-SUN1 α1 is a 144 kDa trimer that dissociates into 100 kDa dimers and 50 kDa monomers (theoretical—148 kDa, 99 kDa and 49 kDa). Data were collected at protein concentrations of 20 mg/mL (red) and 1 mg/mL (grey). **(B)** Spectrophotometric determination of zinc content for wild-type (black; 1.28 Zn^2+^ per trimer) and C376A (red; 0.05 Zn^2+^ per trimer) SUN1 α1, using metallochromic indicator PAR, with zinc standards shown in a gradient from light to dark grey (0–100 µM). **(C and D)** SEC-MALS analysis of **(C)** MBP-SUN1 α1 (20 mg/mL) and **(D)** MBP-SUN1 α1 C376A (5 mg/mL) after purification (black), and after over-night incubation with 2 mM zinc acetate (red) or 10 mM EDTA (yellow). **(C)** MBP-SUN1 α1 is stabilised as a 145 kDa trimer by zinc incubation and is disrupted to a 50 kDa monomer by EDTA. **(D)** MBP-SUN1 α1 C376A is restricted to a 47 kDa monomer in all conditions. **(E)** Structure of designed parallel trimeric coiled-coil Coil Ser L9C bound to arsenic, showing its trigonal coordination by cysteine residues C9 (PDB accession 2JGO; Touw et al., 2007). **(F and G)** SUN1 α1 structure at the central 376-CHHH-379 motif for the **(F)** crystal structure in which one cysteine residue is partially oxidised to S-hydroperoxycysteine and **(G)** modelled structure in which three reduced cysteine residues and a water molecule mediate tetrahedral coordination of a zinc ion.

We next utilised size-exclusion chromatography multi-angle light scattering (SEC-MALS) to assess the oligomeric state of SUN1 α1 in solution. SEC-MALS analysis of an MBP fusion (used to provide greater molecular mass resolution) confirmed that SUN1 α1 is predominantly trimeric (144 kDa), in keeping with our crystallographic and molecular dynamics analyses (Figure 4A). However, we also observed concentration-dependent dissociation in solution, through a 100 kDa dimeric intermediate, to a 50 kDa monomeric species (Figure 4A).

We wondered whether the unusual properties of consecutive cysteine and histidine residues within the central 376-CHHH-379 motif may contribute to stability of the coiled-coil trimer through metal coordination. Using a spectrophotometric 4-(2-pyridylazo) resorcinol assay, we detected the presence of a divalent cation bound to SUN1 α1, at a level consistent with one zinc ion per trimer (Figure 4B). Further, the SUN1 α1 trimer was stabilised by addition of zinc prior to SEC-MALS, and was largely disrupted to a monomer by prior incubation with chelating agent EDTA (Figure 4C). We reasoned that zinc-binding likely involves the conserved cysteine residue of the 376-CHHH-379 motif (Figure 2B). Accordingly, introduction of point mutation C376A eliminated zinc-binding and blocked trimerization, restricting SUN1 α1 to a monomer (Figures 4B, D). Hence, our data suggest that SUN1 α1 trimer is stabilised in solution by zinc-binding to cysteine residue C376.

How can we rationalise stabilisation of the SUN1 α1 trimer by zinc-binding? We observed several cases in the literature in which metal ions are located along the three-fold axis of trimeric coiled-coils (Touw et al., 2007; Zastrow and Pecoraro, 2014; Cristie-David and Marsh, 2019). In one case, arsenic was trigonally coordinated by symmetric cysteine residues (PDB accession 2JGO; Figure 4E), and it was speculated that zinc could be tetrahedrally coordinated through the same arrangement of cysteine residues with water acting as a fourth exogenous ligand (Touw et al., 2007). We reasoned that this coordination pattern may explain zinc-binding by SUN1 α1. Hence, we built a zinc-bound model by changing the rotamer state of cysteine residues to that of metal-bound structures, and positioning zinc and water along the three-fold axis (Figures 4F, G). In the resultant energy-minimised model, the bond lengths and angles between zinc and its cysteine and water ligands closely match those of tetrahedral geometry (Figure 4G). Further, H379 residues of the 376-CHHH-379 motif have a suitable location to complete tetrahedral binding of the water molecule (Figure 4G). Thus, we propose that tetrahedral coordination of zinc by cysteine and water ligands provides the structural basis for stabilisation of the SUN1 α1 trimer by zinc-binding.

### SUN1’s α1 and α2 coiled-coil domains mutually reinforce its trimerization

What is the role of SUN1’s α1 trimeric coiled-coil within its wider luminal structure? We utilised SEC-MALS and size-exclusion chromatography small-angle X-ray scattering (SEC-SAXS) to determine the oligomeric states and structures formed by SUN1 luminal constructs in solution, alongside circular dichroism (CD) to assess their helicity and thermal stability.

**FIGURE 5:**
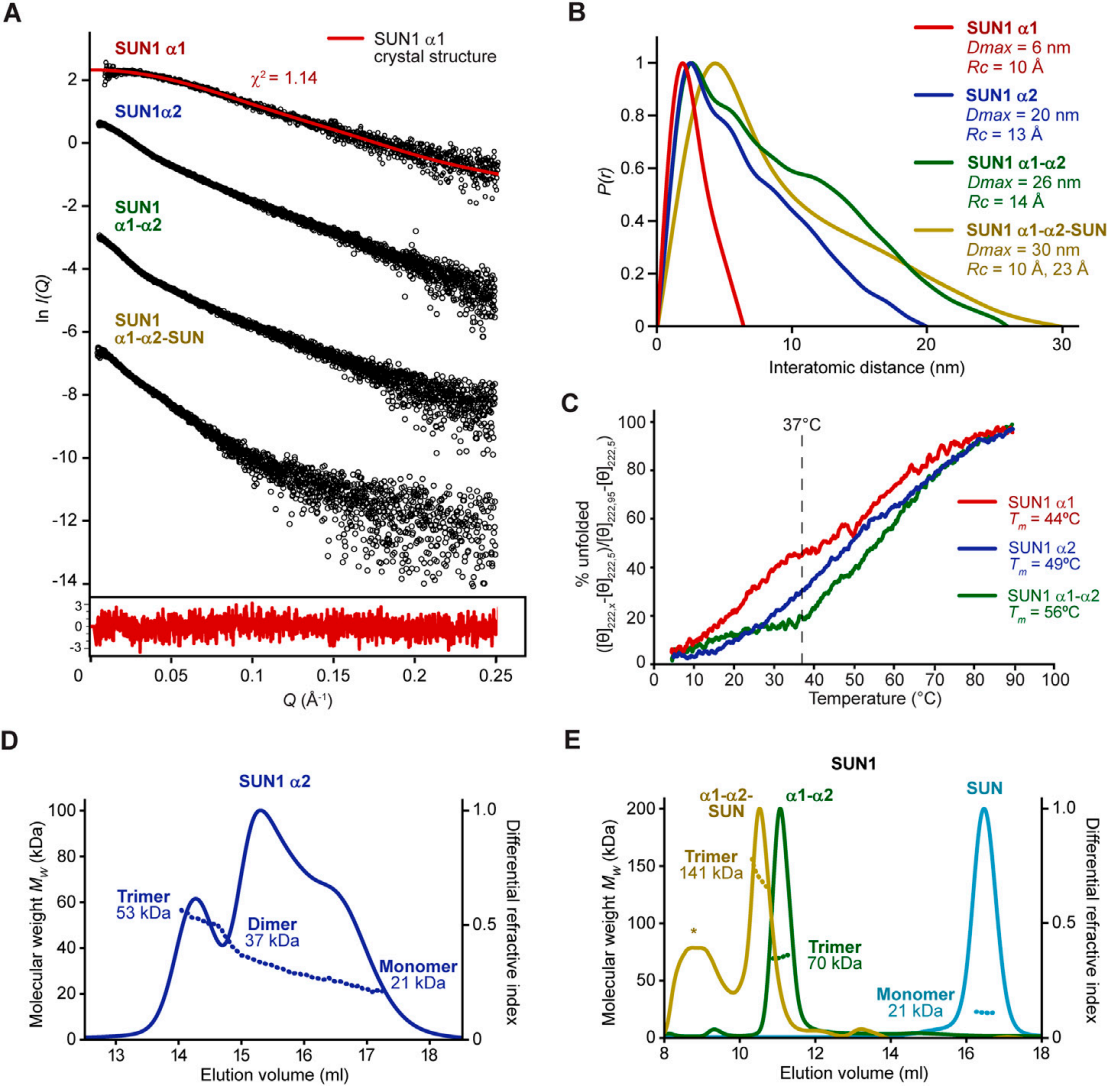
The SUN domain is trimerized by α1 and α2 luminal coiled-coils. **(A and B)** SEC-SAXS analysis of SUN1 α1 (red), α2 (blue), α1-α2 (green) and α1-α2-SUN (yellow). **(A)** SAXS scattering data in which the SUN1 α1 scattering cure is overlaid with the theoretical scattering curve of the SUN1 α1 trimeric coiled-coil crystal structure, showing a χ^2^ value of 1.14. The residuals for the fit are shown (inset). **(B)** SAXS *P(r)* interatomic distance distributions in which maximum dimensions (*Dmax*) are indicated, along with cross-sectional radii (*Rc*) determined from Guinier analysis (Supplementary Figure S4). **(C)** Thermal denaturation recording the circular dichroism (CD) helical signature at 222 nm between 5°C and 95°C, as % unfolded. Melting temperatures were estimated, as indicated. CD spectra are shown in Supplementary Figure S5. **(D and E)** SEC-MALS analysis. **(D)** SUN1 α2 is a 53 kDa trimer that dissociates into 37 kDa dimers and 21 kDa monomers (theoretical—57 kDa, 38 kDa and 19 kDa). **(E)** SUN1 α1-α2-SUN and α1-α2 are 141 kDa and 70 kDa trimers (theoretical—142 kDa and 75 kDa), whereas the isolated SUN domain is a 21 kDa monomer (theoretical—22 kDa). The additional peak marked with an asterisk in the α1-α2-SUN trace corresponds to a wide range of higher molecular weight species of between 0.2–2.0 MDa, so likely represents a non-specific aggregate.

The SUN1 α1 trimer showed SAXS data and corresponding real-space *P(r)* pair-distance distribution function indicating an elongated molecule of approximately 6 nm in length (Figures 5A, B and Supplementary Figure S4A). This matches the 6 nm length of the SUN1 α1 crystal structure. Further, Guinier analysis indicated a cross-sectional radius of 10 Å (Supplementary Figure S4B). We previously established that dimeric coiled-coils have Guinier cross-sectional radii of 8–9 Å, whereas four-helical coiled-coils have cross-sectional radii of 10–14 Å (Dunce et al., 2018a; Dunne and Davies, 2019; Sanchez-Saez et al., 2020; Dunce et al., 2021). Hence, a 10 Å cross-sectional radius is consistent with SUN1 α1 being a trimeric coiled-coil. Finally, the SAXS scattering curve was closely fitted by the SUN1 α1 trimeric coiled-coil structure (χ^2^ = 1.14; Figure 5A). Further, in agreement with our SEC-MALS data indicating concentration-dependent dissociation (Figure 4A), CD showed that SUN1 α1 underwent gradual non-cooperative unfolding, retaining only 55% of its α-helical structure at 37°C, with an arbitrary melting temperature of 44°C (Figure 5C and Supplementary Figure S5). Hence, our combined SEC-MALS, SEC-SAXS and CD data indicate that whilst the SUN1 α1 crystal structure represents the *bona fide* trimeric solution state, it has the propensity to dissociate and unfold at low protein concentrations and high temperatures, consistent with a low micromolar affinity.

We wondered whether the α1 coiled-coil domain may be afforded additional stability by downstream coiled-coils within SUN1’s luminal region. On the basis of conservation and secondary structure prediction (Figure 2B), we identified a second luminal coiled-coil domain (amino-acids 421–584; herein referred to as α2), which was stable in solution following recombinant expression (Supplementary Figure S1). This SUN1 α2 coiled-coil domain includes a region of sequence similarity (less than 20% sequence identity) with SUN2’s CC1 trimeric coiled-coil (Nie et al., 2016), and is separated from the α1 coiled-coil domain by a predicted unstructured sequence of 22 amino-acids (Figure 2B). SEC-MALS analysis of SUN1 α2 revealed a trimeric structure (53 kDa) that underwent dissociation, through a 37 kDa dimeric intermediate, to a 21 kDa monomer (Figure 5D). SEC-SAXS analysis of the trimer indicated maximum dimensions and cross-sectional radius compatible with it forming a trimeric coiled-coil (Figures 5A,B and Supplementary Figures S4C, D). Further, CD showed a gradual non-cooperative pattern of unfolding, retaining approximately 70% of its α-helical structure at 37°C, with an arbitrary melting temperature of 49°C (Figure 5C and Supplementary Figure S5). Thus, α1 and α2 luminal coiled-coil domains similarly form trimers that dissociate, so are predicted to be dynamic in solution and potentially within their cellular context.

We next tested how the α1 and α2 coiled-coils behave together when joined by the intervening 22 amino-acid sequence (amino-acids 362–584; herein referred to as α1-α2). SUN1 α1-α2 was soluble following recombinant expression (Supplementary Figure S1), and SEC-MALS analysis showed that it forms a stable trimer, with no dissociation to lower oligomeric species (Figure 5E). Further, CD showed cooperative unfolding, with retention of over 80% of its α-helical structure at 37°C, and a melting temperature of 56°C (Figure 5C and Supplementary Figure S5). It is unlikely that the intervening 22 amino-acid linker mediates formation of a single continuous α1-α2 trimeric coiled-coil as its sequence, which includes four glycine and three proline residues, is strongly predicted to be unstructured. Instead, we propose that α1 and α2 coiled-coil domains are flexibly linked, mutually reinforcing their trimeric structure, and thereby stabilising trimerization of the whole luminal coiled-coil region. This is supported by its SEC-SAXS dimensions, which are consistent with a linear arrangement of α1 and α2 trimeric coiled-coils (Figures 5A,B and Supplementary Figure S4E, F). Finally, we analysed SUN1’s entire structured luminal domain (amino-acids 362–785; herein referred to as α1-α2-SUN), confirming that the stable and non-dissociating trimeric structure was retained upon inclusion of its C-terminal SUN domain (Figure 5E). Hence, SUN1’s α1 and α2 domains have mutually reinforcing trimeric coiled-coil structures that combine to hold together three SUN domains at the end of a luminal trimer.

### Molecular model for the SUN1 luminal structure

**FIGURE 6:**
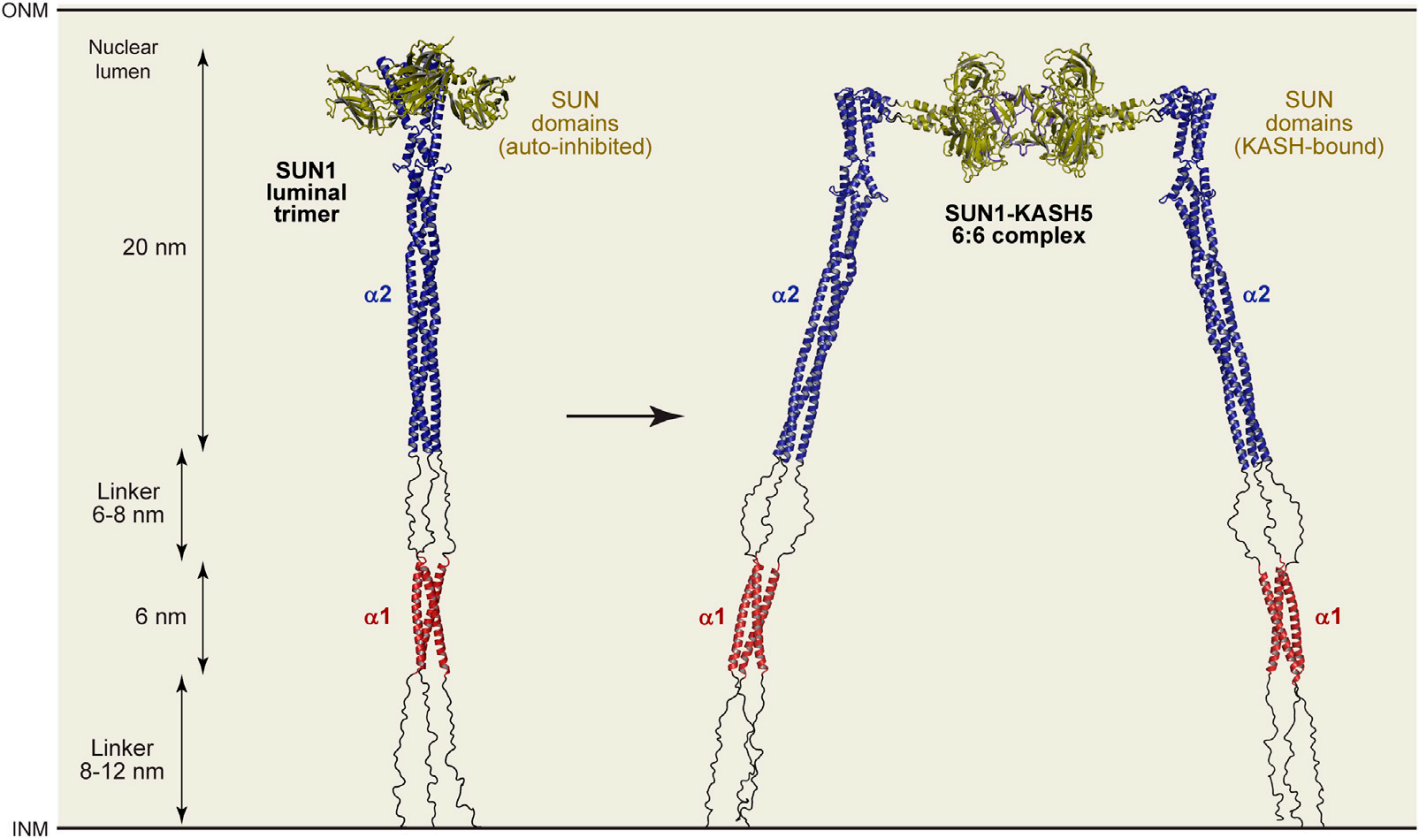
Structure-directed models of luminal SUN1 and SUN1-KASH5 complexes. Models of the SUN1 luminal trimer (left) and the SUN1-KASH5 luminal 6:6 complex (right), based on structures and Alphafold2 multimer models generated using specified templates. Modelling details are shown in Supplementary Figures S6-S8. The structured regions of both conformations have lengths of 6 nm and 20 nm, whereas intervening linkers may adopt relaxed linear (as shown) or stretched conformations, varying between lengths of 8-12 nm and 6-8 nm. Hence, the overall length of the SUN1 luminal trimer and SUN1-KASH5 6:6 complex is predicted to vary between 40-46 nm depending on the magnitude and direction of applied tension forces. For comparison, Alphafold2 multimer predictions of the SUN1 trimer without the use of templates, the full 6:6 complex using templates, and the core 6:6 complex without templates, are shown in Supplementary Figures S9A-I, respectively.

To integrate our findings with those of previous studies, we built a structure-directed model of SUN1’s luminal region using a local installation of *Alphafold2* multimer (Evans et al., 2021; Jumper et al., 2021) in which we could direct its use of structural templates. We specified the use of our SUN1 α1 structure (PDB accession 8AU0), the SUN2 CC1 structure (PDB accession 5ED9; Nie et al., 2016) and the autoinhibited SUN domain structure (PDB accession 5YWZ; Xu et al., 2018) as templates for modelling SUN1’s α1, α2 and SUN domains. The resultant trimeric models of SUN1’s luminal region (amino-acids 326–785) were consistent, with high pLDDT and low PAE scores in structured regions. The pLDDT score is the per-residue confidence in the local surrounding structure within each chain, whereas PAE is predicted aligned error between distant regions within and between chains of multimers (Evans et al., 2021; Jumper et al., 2021). We selected the top-ranked model, and extended its unstructured linkers to present this in a ‘relaxed’ linear state (Figure 6 and Supplementary Figure S6). In the model, the first 33 amino-acids (after the transmembrane region) are unstructured, consistent with their poor conservation, lack of secondary structure prediction and presence of four proline residues (Figure 1B). The subsequent α1 trimer structure is linked to a modelled α2 trimer by a 22 amino-acid unstructured/flexible linker, in keeping with our previous analysis. The α2 trimeric coiled-coil is continuous with helices at the beginning of the SUN domains, which are maintained in autoinhibited conformations, in three-fold symmetry, oriented away from the coiled-coil axis (Figure 6). Whilst this model must be considered as a prediction, it provides a molecularly plausible explanation for the architecture of SUN1 within the nuclear lumen. Further, it allows a means for estimating the maximum length that could be bridged by SUN1 molecules. The α1 and α2 structures are approximately 6 nm and 20 nm long, matching the lengths determined by SEC-SAXS analysis (Figures 5A, B and Supplementary Figures S4A–D). The two flexible linkers can vary in length between approximately 8–12 nm and 6–8 nm, depending on whether they are relaxed or at full stretch. Hence, we predict relaxed lengths of up to 32 nm for both α1-α2 and α1-α2-SUN constructs (which lack the first flexible linker), matching the 26 nm and 30 nm lengths determined by SEC-SAXS analysis (Figures 5A, B and Supplementary Figures S4E–H). Hence, our molecular model for the SUN1 luminal trimer agrees with its experimentally determined dimensions. In total, the full SUN1 luminal trimer is predicted to be between 40–46 nm (Figure 6), depending on tension forces, consistent with the nuclear luminal width (Watson, 1955).

### Molecular model for the luminal meiotic LINC complex

Finally, we used the same technique to build a model of the entire luminal region of the SUN1-KASH5 meiotic LINC complex. We first modelled a SUN1 α2-SUN complex (amino-acids 421–785), with SUN domains in trimeric conformation, using as structural templates the SUN2 CC1 crystal structure (PDB accession 5ED9; Nie et al., 2016) and the SUN1-KASH5 structure (PDB accession 6R2I; Gurusaran and Davies, 2021), but not autoinhibited SUN domains. As previously, models were consistent, with high pLDDT and low PAE scores within structured regions (Supplementary Figure S7). We combined this model with the SUN1-KASH5 6:6 core structure (PDB accession 6R2I; Gurusaran and Davies, 2021), and the N-terminal region of the previous SUN1 luminal trimer model, to model the entire SUN1-KASH5 luminal 6:6 complex (Figure 6 and Supplementary Figure S8). In this model, SUN domains adopt their trimeric KASH-bound conformation, in a “flower-like” arrangement of SUN domains around the coiled-coil stem. Interestingly, α2 and SUN domains are joined by a short flexible linker, which corresponds to a helix-loop-helix turn of the autoinhibited conformation. Hence, the SUN1-KASH5 interface is flexibly oriented relative to the coiled-coil, providing an explanation for how SUN1 transitions from being perpendicular to parallel to the nuclear membrane for KASH-binding. Further even with a 90° bend between α2 and SUN domains, this structure is approximately the same length as the SUN1 luminal trimer, so is also predicted to stretch between 40–46 nm, in keeping with the width of the nuclear lumen (Watson, 1955). Thus, we conclude by presenting a model of the luminal region of the meiotic LINC complex (Figure 6), demonstrating how all existing structural information can be integrated into a molecularly plausible structure that fulfils the necessary geometrical requirements for force transduction between inner and outer nuclear membranes within a 6:6 head-to-head LINC complex assembly.

## Discussion

The LINC complex operates over a cellular scale, bridging between the cytoskeleton and nuclear contents across lengths of potentially hundreds of nanometres, but is formed principally of coiled-coils that are less than 2 nm in width. Hence, the LINC complex falls within a “grey area” of biology, in which the scale of its full assembly is too large for high resolution methods, but its smallest dimensions require higher resolutions than can be achieved by cellular microscopy (Joseph et al., 2017; Goodsell et al., 2020). Thus, to understand its structure requires an integrative approach in which we combine high-resolution structures of domains *in silico* to obtain models that explain its molecular structure at a biological scale. We have integrated our crystal structure of SUN1’s luminal α1 coiled-coil domain with previous structures and biophysical data to build molecular models of luminal SUN1 and the LINC complex at a scale relevant to the nuclear luminal width (Watson, 1955). Thus, we have provided the first full molecular model of the luminal architecture of the LINC complex.

The SUN1 α1 trimer was stabilised by zinc-binding, which we modelled as tetrahedral coordination involving C376 residues, in keeping with previous metal-bound trimeric coiled-coil structures (Touw et al., 2007; Zastrow and Pecoraro, 2014; Cristie-David and Marsh, 2019). However, the zinc-bound trimer demonstrated a propensity for dissociation in solution, and incubation with exogenous zinc was required to enhance its stability. Further, the crystal structure lacked bound zinc, but included a partially oxidised cysteine residue, which seemingly provided an alternative means for structural stabilisation. Thus, the SUN1 α1 trimer appears to be dynamic, raising the possibility that its assembly could be regulated by the availability of zinc, or another divalent cation, within the nuclear lumen. Further, the nature of the central 376-CHHH-379 motif suggests that cysteine oxidation, disulphide formation and protonation could also affect assembly. Indeed, zinc-binding and cysteine oxidation are mutually exclusive, so oxidation could provide a means for irreversibly blocking zinc-induced trimerization. Similarly, disulphide bond formation between C376 residues could stabilise a dimeric conformation. Such regulatory mechanisms have previously been proposed based on observations that SUN2’s luminal trimer is disrupted by low pH and calcium, (Jahed et al., 2018b), the SUN-KASH interaction is enhanced by calcium (Majumder et al., 2022), and that SUN1 trimers may be linked together by inter-molecular disulphide bond formation (Lu et al., 2008). Nevertheless, the biological roles of zinc-binding and other hypothesised mechanisms remain unknown, and must be determined experimentally *in vivo*.

**FIGURE 7:**
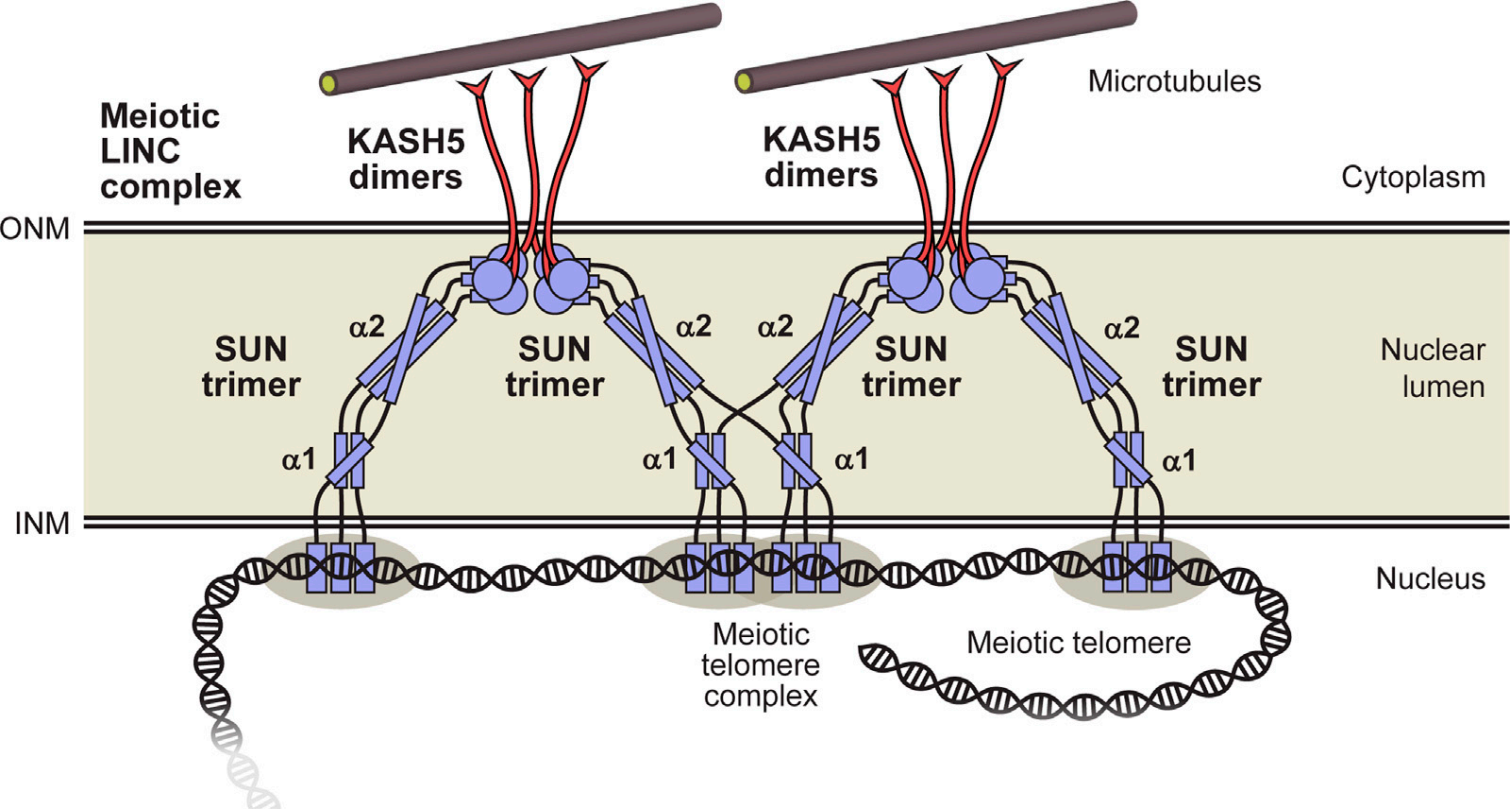
Model for the luminal structure of SUN1 within the meiotic LINC complex. The luminal region of SUN1 consists of α1 and α2 trimeric coiled-coil domains, and a C-terminal globular SUN domain that interact head-to-head within 6:6 complexes with KASH5 proteins. These discrete domains are linked together, and to the transmembrane region (at the INM), by flexible unstructured sequences. These intervening flexible linkers provide the possibility for domain-swap interactions between adjacent 6:6 LINC complexes that my contribute to branching within a force-transducing meiotic LINC complex network. Additional components, such as dynein and dynactin, are not depicted to enhance clarity.

An important prediction from our structure-directed models is that SUN1’s luminal region consists of three structural units separated by flexible linkers. The α1, α2 and SUN domains are formed by approximately 87% of the 460 amino-acid luminal region, with remaining amino-acids forming flexible linkers. The first linker of 33 amino-acids bridges from the transmembrane region to the α1 coiled-coil, the second linker of 22 amino-acids connects this to the α2 coiled-coil, and then the final linker of 5 amino-acids joins this to the initial coiled-coil of the SUN domain (this linker is absent in the autoinhibited SUN domain conformation). Their flexible nature is indicated by amino-acid composition (including a large proportion of glycine and proline residues), lack of predicted secondary structure and structured-directed *Alphafold2* models. The presence of these flexible linkers suggests that SUN1 does not form a continuous rod-like coiled-coil between nuclear membranes. Instead, it likely forms a string of linked rigid structural units with conformational freedom to move relative to one another within the lumen (Figure 7), in agreement with previously proposed models (Jahed et al., 2021). Our model is based on the SUN-KASH complex having a 6:6 stoichiometry as this is the only oligomeric state that has been observed in crystal structures and in solution (Sosa et al., 2012; Wang et al., 2012; Zhou et al., 2012; Cruz et al., 2020; Gurusaran and Davies, 2021). Nevertheless, our model of the SUN1 luminal region is compatible with other SUN-KASH stoichiometries, such as 3:3 or larger oligomers, which could potentially form in proximity of the outer nuclear membrane *in vivo* (Jahed et al., 2021).

What is the benefit of SUN1’s luminal region consisting of linked coiled-coil domains rather than forming a single continuous coiled-coil? Firstly, the presence of intervening flexible linkers may facilitate coiled-coil folding by overcoming the topological challenge of coiling chains of up to 300 amino-acids around each another. Secondly, conformational freedom between coiled-coil and SUN domains provides a simple explanation for how SUN1 can reorient from perpendicular to parallel to the nuclear membrane as it crosses the nuclear lumen to form SUN-KASH 6:6 complexes (Gurusaran and Davies, 2021). Indeed, the change in helical angulation could be achieved by a single 90° bend in one linker (as shown in the model), or through progressive angulation at each linked step (Figure 7). Finally, linked coiled-coil domains could in principle adapt to changing tension forces, adopting conformations that are more angled or perpendicular to the nuclear membrane in response to forces in these directions. Importantly, our models demonstrate that flexible linkers must be largely stretched for LINC complexes to reach across nuclear widths of >40 nm (Watson, 1955). This is consistent with the need for flexible linkers to bear tension during force transduction by the LINC complex.

We observed that the SUN1 α1 and α2 coiled-coil domains are dissociating oligomers that form stable trimers when joined by their intervening linker. It was previously shown that SUN2’s CC1 coiled-coil domain (corresponding to α2) with subsequent SUN domain also dissociates (Nie et al., 2016; Jahed et al., 2018b), whereas its full luminal region forms a stable trimer (Sosa et al., 2012). Hence our findings may be conserved in SUN2, in which the predicted coiled-coil upstream of CC1 may have a stabilising role analogous to SUN1’s α1 domain. The presence of flexibly linked discrete coiled-coil domains raises the possibility of domain-swap interactions in which α1 and α2 sequences may form coiled-coil structures with chains from different SUN1 molecules (Figure 7). This is unlikely to occur in solution as the proximity of tethered sequences greatly favours the formation of coiled-coils between the same chains. However, we speculate that it may occur *in vivo* if SUN1 molecules are present at a sufficiently high local concentration for upstream interactions to occur with similar likelihood between chains of the same or distinct downstream trimers. This has two potentially beneficial consequences. Firstly, dissociation and reassociation with different chains may overcome tangles that could develop as the luminal structure adapts to altering tension forces and structural changes. Secondly, domain-swap interactions may facilitate force propagation by providing branch sites within the LINC complex axis. Hence, they may contribute to the force integration and distribution provided by oligomer state alteration between KASH5 dimers, SUN1-KASH5 6:6 complexes and SUN1 trimers (Gurusaran and Davies, 2021; Garner et al., 2022), disulphide bond formation between SUN1 trimers (Lu et al., 2008), and other higher-order interactions between SUN proteins (Jahed et al., 2018a), to facilitate force transduction through a branched LINC complex network. This model is consistent with the observation that SUN1’s luminal domain has been shown to form oligomers that are larger than trimers upon expression in cellular systems (Hennen et al., 2018).

Here, we have used the example of the meiotic LINC complex to illustrate the structure-function relationships inherent in LINC complex architecture. Indeed, the transmission of microtubule-generated forces to achieve the meiotic chromosome movements exemplifies the challenges of LINC-mediated force transduction and the necessity for adaptivity, load bearing and distribution by SUN1’s luminal structure (Ding et al., 2007; Horn et al., 2013b). Nevertheless, the LINC complex has several other specialised roles such as in hearing (Horn et al., 2013a), and is essential for nuclear structure, shape and positioning (Crisp et al., 2006; Luxton et al., 2010; Alam et al., 2015; Kozono et al., 2018). Hence, the molecular models for luminal SUN1 and LINC complex architecture presented herein should be directly applicable to the generalised and specialised functions of the LINC complex in its many and varied cellular roles.

## Materials and methods

### Recombinant protein expression and purification

Sequences corresponding to human SUN1 (amino-acids 362–401, 421–584, 362–584, 362–785; Uniprot accession O94901) were cloned into pMAT11 vectors (Peranen et al., 1996) for expression as TEV-cleavable N-terminal His-MBP- fusion proteins. Constructs were expressed in BL21 (DE3) cells (Novagen^®^) in 2xYT media, induced with 0.5 mM IPTG for 16 h at 25°C. Cells were lysed by sonication in 20 mM Tris pH 8, 500 mM KCl, and fusion proteins were purified from clarified lysate through consecutive Ni-NTA (Qiagen), amylose (NEB) and HiTrap Q HP (Cytiva) ion exchange chromatography. Affinity tags were removed by incubation with TEV protease and cleaved samples were purified by HiTrap Q HP ion exchange chromatography and size exclusion chromatography (HiLoad™ 16/600 Superdex 200, Cytiva) in 20 mM HEPES pH 7.5, 150 mM KCl, 2 mM DTT. Protein samples were concentrated using Pall 10 kDa Microsep™ Advance centrifugal devices, except for SUN1 362–401 where Pall 3 kDa Microsep™ Advance centrifugal devices were used, and were stored at −80°C following flash-freezing in liquid nitrogen. Protein samples were analysed by SDS-PAGE with Coomassie staining, and concentrations were determined by UV spectroscopy using a Cary 60 UV spectrophotometer (Agilent) with extinction coefficients and molecular weights calculated by ProtParam (http://web.expasy.org/protparam/).

### Crystallisation and structure solution of SUN1 α1

SUN1 362–401 protein crystals were obtained through vapour diffusion in sitting drops, by mixing 100 nL of protein at 3.5 mg/mL with 100 nL of crystallisation solution (0.09 M Sodium nitrate, 0.09 M Disodium phosphate, 0.09 M Ammonium sulfate, 0.1 M imidazole pH 6.5, 0.1 M MES (acid), 37.5% MPD (racemic), 37.5% PEG 1K, 37.5% PEG 3350) and equilibrating at 20°C for 10–20 days. Crystals were flash frozen in liquid nitrogen. X-ray diffraction data were collected at 0.9795 Å, 100 K, as 2000 consecutive 0.10° frames of 0.040 s exposure on an Eiger2 XE 16 M detector at beamline I04 of the Diamond Light Source synchrotron facility (Oxfordshire, United Kingdom) on 12/05/2019. Data were processed using *AutoPROC* (Vonrhein, 2011), in which indexing, integration, scaling were performed by XDS (Kabsch, 2010) and Aimless (Evans, 2011). Crystals belong to monoclinic spacegroup P2_1_ (cell dimensions a = 31.31 Å, b = 35.99 Å, c = 46.10 Å, *α* = 90°, *β* = 104.54°, *γ* = 90°), with a SUN1 trimer in the asymmetric unit. Data were corrected for anisotropy using the UCLA diffraction anisotropy server (https://services.mbi.ucla.edu/anisoscale/) (Strong et al., 2006), imposing anisotropic limits of 2.4 Å, 2.1 Å, 2.1 Å, with principal components of 19.50 Å^2^, −9.18 Å^2^ and −10.31 Å^2^. Structure solution was achieved through fragment-based molecular replacement using *ARCIMBOLDO_LITE* (Rodriguez et al., 2009), in which six helices of 18 amino acids were placed by *PHASER* (McCoy et al., 2007) and extended by tracing in *SHELXE* utilising its coiled-coil mode (Caballero et al., 2018). A correct solution was identified by a *SHELXE* correlation coefficient of 52.9%. Model building was performed through iterative re-building by *PHENIX* Autobuild (Adams et al., 2010) and manual building in *Coot* (Emsley et al., 2010). Additional density was observed for cysteine residue C376 of chain B, which was modelled as alternative conformations of a reduced cysteine and peroxysulfenic acid (2CO), which refined to occupancies of 0.38 and 0.62, respectively. The structure was refined using *PHENIX* refine (Adams et al., 2010), using isotropic atomic displacement parameters, against anisotropy-corrected 2.07 Å data, to R and R_free_ values of 0.2438 and 0.2551 respectively, with 100% of residues within the favoured regions of the Ramachandran plot (0 outliers), clashscore of 6.38 and overall *MolProbity* score of 1.35 (Chen et al., 2010). The final SUN1 model was analysed using the *Online_DPI* webserver (http://cluster.physics.iisc.ernet.in/dpi) to determine a Cruikshank diffraction precision index (DPI) of 0.25 Å (Kumar et al., 2015).

### Molecular dynamics

Molecular dynamics (MD) simulations were performed using *AMBER* ff19SB and OPC forcefields (Case et al., 2022) in *OpenMM* (Eastman et al., 2017), run locally on NVIDIA GeForce RTX 3090 GPU cards through a Google Colab notebook that was modified from the “Making-it-rain” cloud-based MD notebook (Arantes et al., 2021). The SUN1 trimer was placed in a water box 10 Å larger than the structure, and was neutralised at a KCl concentration of 150 mM, by *AMBER* tleap (Case et al., 2022). The structure was equilibrated for 200 ps, and then run for 1 μs at 310 K and 1 bar pressure, using periodic boundary conditions, with the Langevin Middle Integrator and MonteCarlo Barostat, with integration times of 2 fs The run was repeated three times. MD trajectories were analysed using *pytraj* (Roe and Cheatham, 2013; Hai Nguyen et al., 2016).

### Size-exclusion chromatography multi-angle light scattering (SEC-MALS)

The absolute molar masses of SUN1 protein samples were determined by multi-angle light scattering coupled with size exclusion chromatography (SEC-MALS). SUN1 protein samples at > 5 mg/mL (unless otherwise stated) were loaded onto a Superdex™ 200 Increase 10/300 GL size exclusion chromatography column (Cytiva) in 20 mM HEPES pH 7.5, 150 mM KCl, 2 mM DTT, at 0.5 mL/min, in line with a DAWN^®^ HELEOS™ II MALS detector (Wyatt Technology) and an Optilab^®^ T-rEX™ differential refractometer (Wyatt Technology). For induction and disruption of zinc-binding, samples were pre-incubated with 2 mM zinc acetate or 10 mM EDTA overnight prior to analysis. Differential refractive index and light scattering data were collected and analysed using ASTRA^®^ 6 software (Wyatt Technology). Molecular weights and estimated errors were calculated across eluted peaks by extrapolation from Zimm plots using a dn/dc value of 0.1850 mL/g.

### Spectrophotometric determination of zinc content

The presence of zinc in protein samples was determined through a spectrophotometric method using the metallochromic indicator 4-(2-pyridylazo) resorcinol (PAR) (Sabel et al., 2009). Protein samples at 70 μM, corresponding to SUN1 α1 wild-type and C376A, were digested with 0.6 μg/μL proteinase K (NEB) at 60°C for 1 h. Of the supernatant, 10 μL of each protein digestion was added to 80 μL of 50 μM 4-(2-pyridylazo)-resorcinol (PAR) in 20 mM Tris, pH 8.0, 150 mM KCl, incubated for 5 min at room temperature, and UV absorbance spectra were recorded between 600 and 300 nm (Varian Cary 60 spectrophotometer). Zinc concentrations were estimated from the ratio between absorbance at 492 and 414 nm, plotted on a line of best fit obtained from analysis of 0–100 μM zinc acetate standards.

### Size-exclusion chromatography small-angle X-ray scattering (SEC-SAXS)

SEC-SAXS experiments were performed at beamline B21 of the Diamond Light Source synchrotron facility (Oxfordshire, United Kingdom). Protein samples at concentrations >5 mg/mL were loaded onto a Superdex™ 200 Increase 10/300 GL size exclusion chromatography column (Cytiva) in 20 mM HEPES pH 7.5, 150 mM KCl at 0.5 mL/min using an Agilent 1200 HPLC system. The column outlet was fed into the experimental cell, and SAXS data were recorded at 12.4 keV, detector distance 4.014 m, in 3.0 s frames. Data were subtracted and averaged, and analysed for Guinier region *Rg* and cross-sectional *Rg* (*Rc*) using *ScÅtter* 4.0 (http://www.bioisis.net), and *P(r)* distributions were fitted using *PRIMUS* (Konarev et al., 2003). Crystal structures and models were fitted to experimental data using *CRYSOL* (Svergun and Koch, 1995).

### Circular dichroism (CD) spectroscopy

Far UV circular dichroism (CD) spectroscopy data were collected on a Chirascan VX CD spectrometer (School of Chemistry, University of Edinburgh). CD spectra were recorded in 10 mM Na_2_HPO_4_/NaH_2_PO_4_ pH 7.5, 150 mM NaF, at protein concentrations between 0.1–0.3 mg/mL, using a 0.5 mm pathlength quartz cuvette (Applied Photophysics), at 0.2 nm intervals between 260 and 185 nm at 4°C. Spectra were averaged across three accumulations, corrected for buffer signal, smoothed and converted to mean residue ellipticity ([θ]) (x1,000 deg. cm^2^. dmol^−1^. residue^−1^). CD thermal denaturation was performed in 10 mM Na_2_HPO_4_/NaH_2_PO_4_ pH 7.5, 150 mM NaF, at protein concentrations between 0.1–0.3 mg/mL, using a 0.5 mm pathlength quartz cuvette (Applied Photophysics). Data were recorded at 222 nm, between 4°C and 95°C, at 0.5°C intervals with ramping rate of 2°C per minute, and were converted to mean residue ellipticity ([θ_222_]) and plotted as % unfolded ([θ]_222,x_-[θ]_222,5_)/([θ]_222,95_-[θ]_222,5_). Melting temperatures (Tm) were estimated as the points at which samples are 50% unfolded.

### SUN1 and SUN1-KASH5 luminal structural modelling

Models were generated using a local installation of *Alphafold2* v2.2.2 (Jumper et al., 2021). This installation was modified to control the use of templates from the PDB and allow additional templates from newly solved crystal structures. Models of the SUN1 luminal trimer (amino-acids 326–785) were generated through the multimer pipeline (Evans et al., 2021), using PDB structures 5YWZ (Xu et al., 2018), 5ED9 (Nie et al., 2016) and the newly reported α1 crystal structure 8AU0, as the sole templates. The constituent α1 and α2-SUN domains of the resultant model were re-positioned in line, and their intervening linkers were re-modelled in “relaxed” linear conformations. For the model of the meiotic SUN1-KASH5 luminal LINC complex, the α2-SUN trimer (amino-acids 421–785) was first modelled in trimeric SUN domain conformation by the *Alphafold2* multimer pipeline (Evans et al., 2021; Jumper et al., 2021), using PDB structures 5ED9 (Nie et al., 2016) and 6R2I (Gurusaran and Davies, 2021) as the sole templates. The SUN domain trimer of the resultant structure was replaced with one KASH5-bound trimer of the SUN1-KASH5 6:6 core complex structure (PDB accession 6R2I; Gurusaran and Davies, 2021), with re-modelling of the intervening flexible linkers. The structure was combined with the α1 domain flanked by flexible linkers of the previous SUN1 luminal trimer model, and was replicated for the second KASH5-bound trimer of the complex, to achieve a full model of the SUN1-KASH5 luminal 6:6 structure. *Alphafold2* multimer modelling data were analysed using modules from the ColabFold notebook (Mirdita et al., 2022). Models were edited, combined and flexible linkers were remodelled using the *PyMOL* Molecular Graphics System, Version 2.0.4 Schrödinger, LLC, and *Coot* (Emsley et al., 2010).

### Protein sequence and structure analysis

Multiple sequence alignments were generated using *Jalview* (Waterhouse et al., 2009), and molecular structure images were generated using the *PyMOL* Molecular Graphics System, Version 2.0.4 Schrödinger, LLC.

### Statistics and reproducibility

All biochemical and biophysical experiments were repeated at least three times with separately prepared recombinant protein material. Molecular dynamics simulations were performed in triplicate by repeating every step of the simulation from the same structural model.

## Data Availability

Crystallographic structure factors and atomic co-ordinates have been deposited in the Protein Data Bank (PDB) under accession number 8AU0, and corresponding raw diffraction images have been deposited at https://proteindiffraction.org/.
