# Supplementary material for: Molecular insights into LINC complex architecture through the crystal structure of a luminal trimeric coiled-coil domain of SUN1
Source: Front Cell Dev Biol. 2023 Jun 21;11:1144277. doi: 10.3389/fcell.2023.1144277 (PMC10320395; doi:10.3389/fcell.2023.1144277)
Supplement: Supplementary file 1 [file Presentation1.pdf]

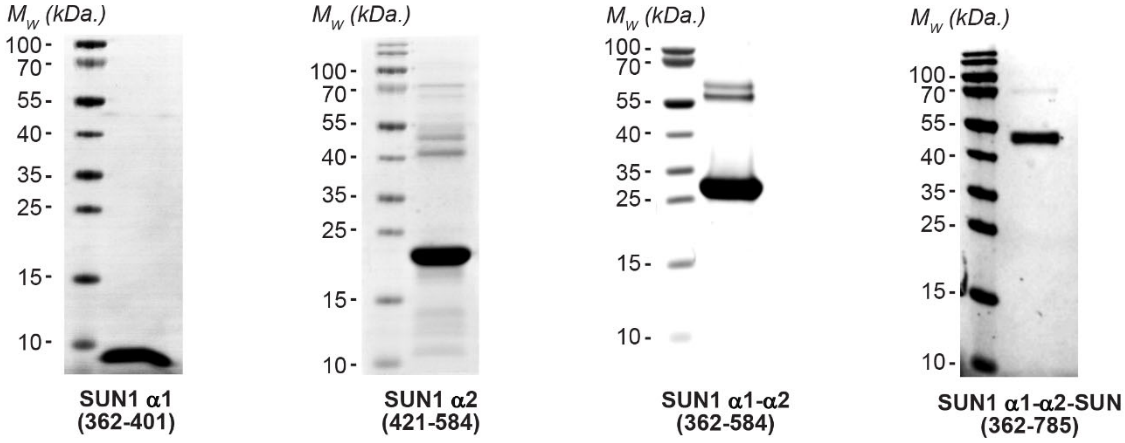

Supplementary Figure S1

SDS-PAGE of protein samples used in this study.

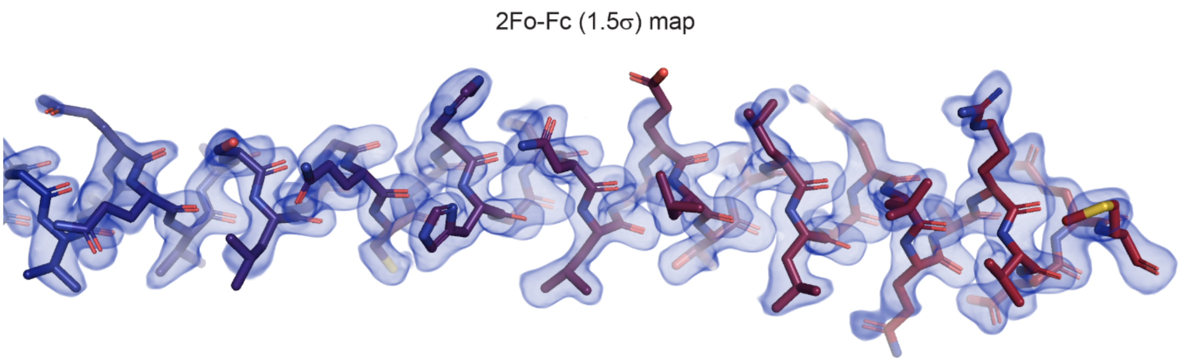

Supplementary Figure 2

Crystal structure of the SUN1 α1 trimer.

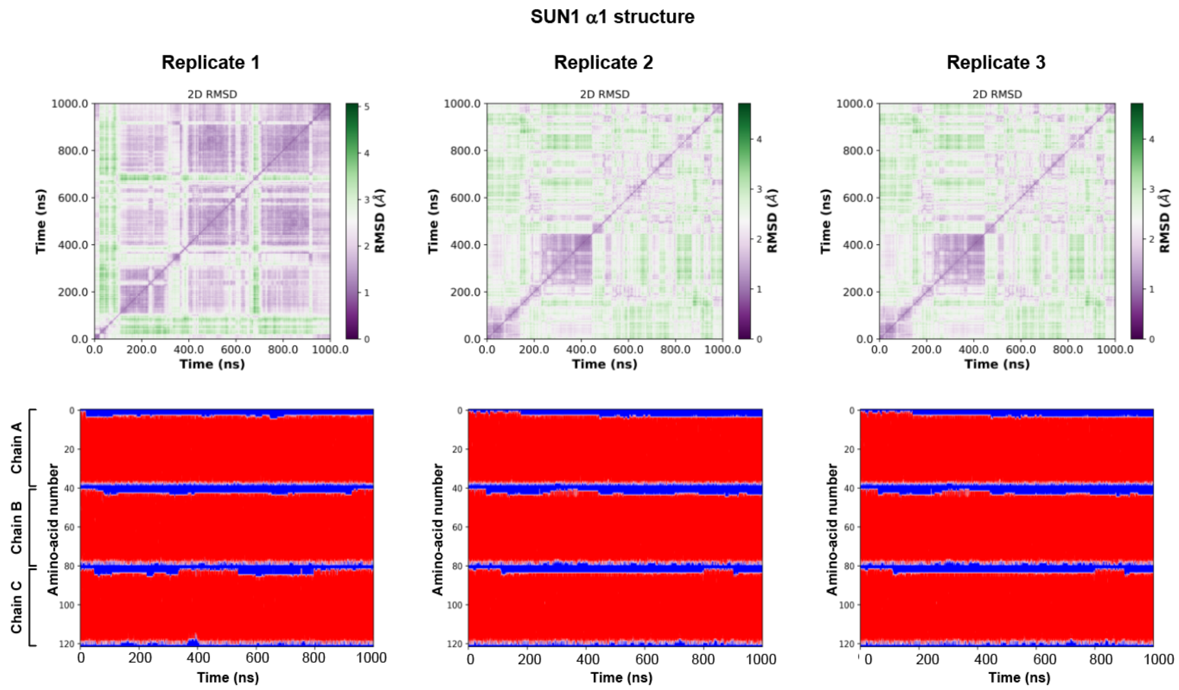

Supplementary Figure S3

Molecular dynamics simulations of the SUN1 α1 trimer.

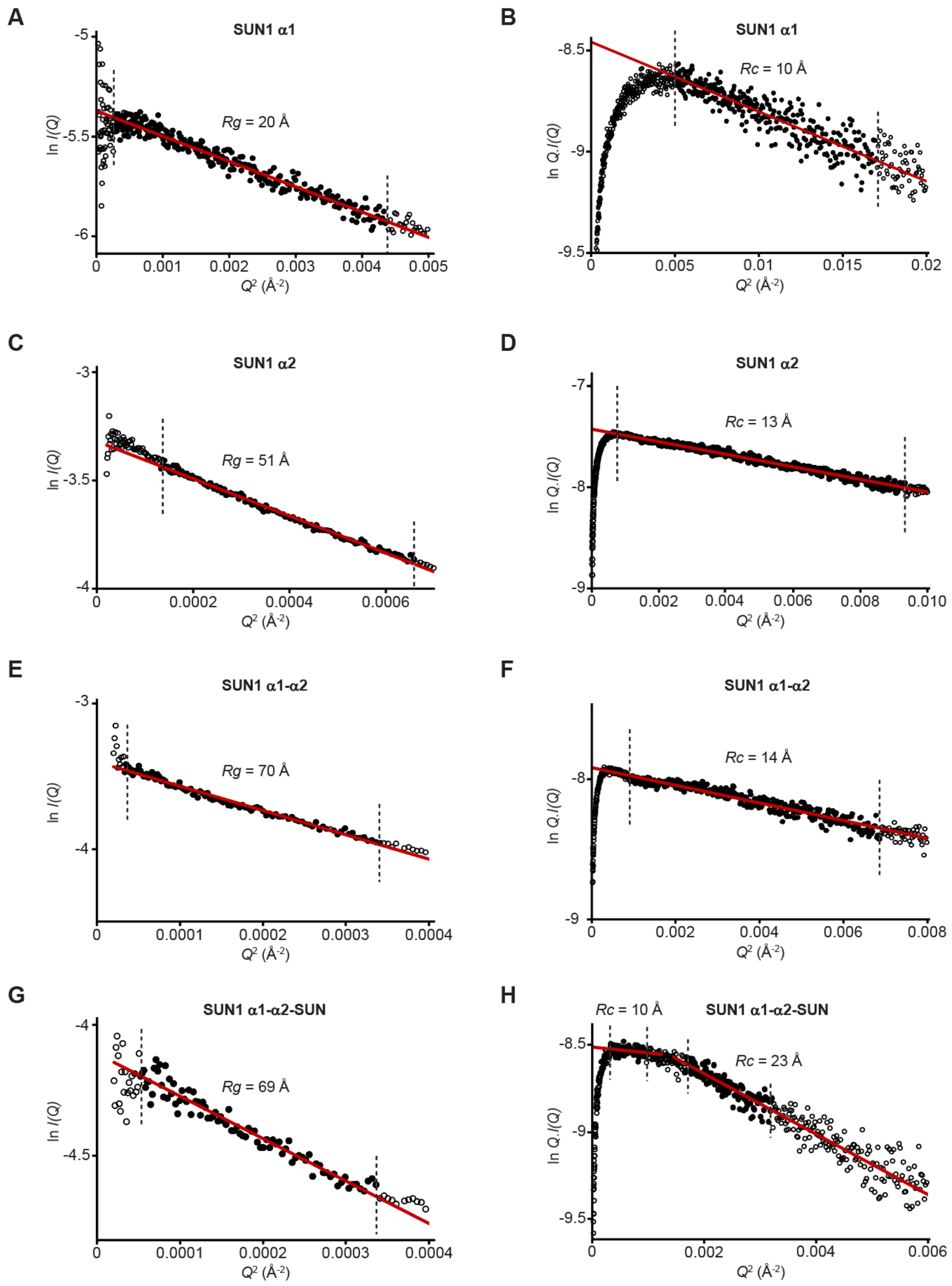

Supplementary Figure S4

Solution SEC-SAXS analysis of the SUN1 luminal constructs.

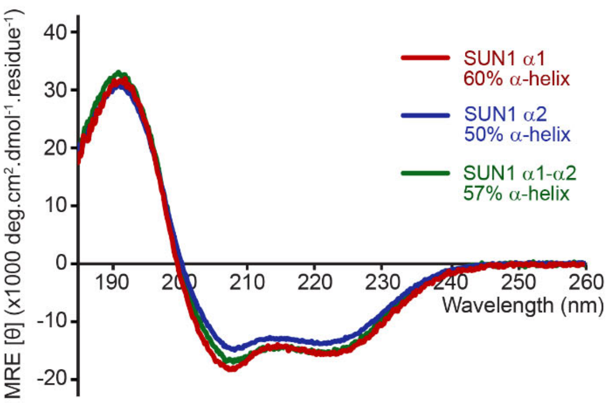

Supplementary Figure S5

Circular dichroism (CD) analysis.

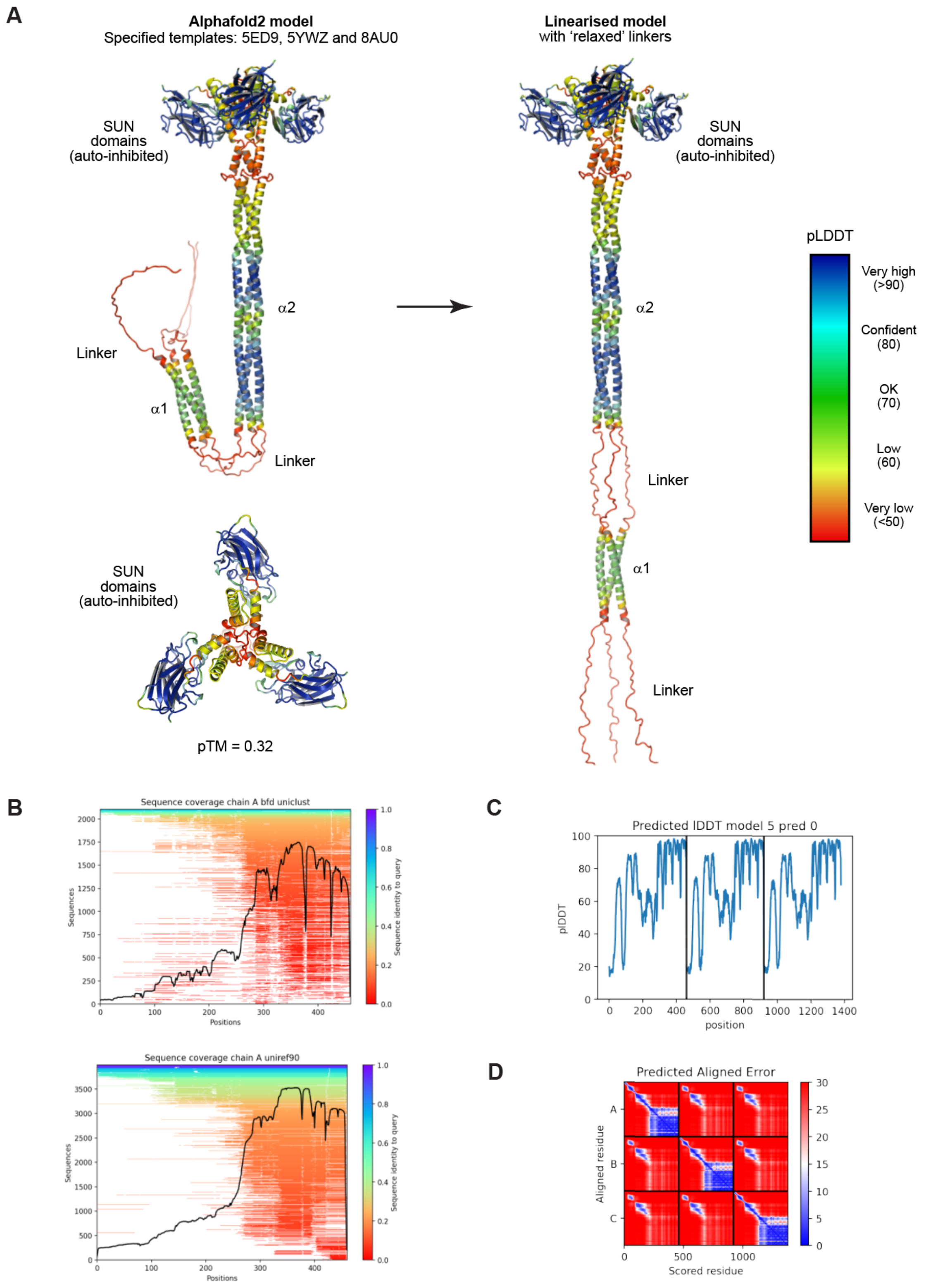

Supplementary Figure S6

Modelling of the SUN1 luminal trimer.

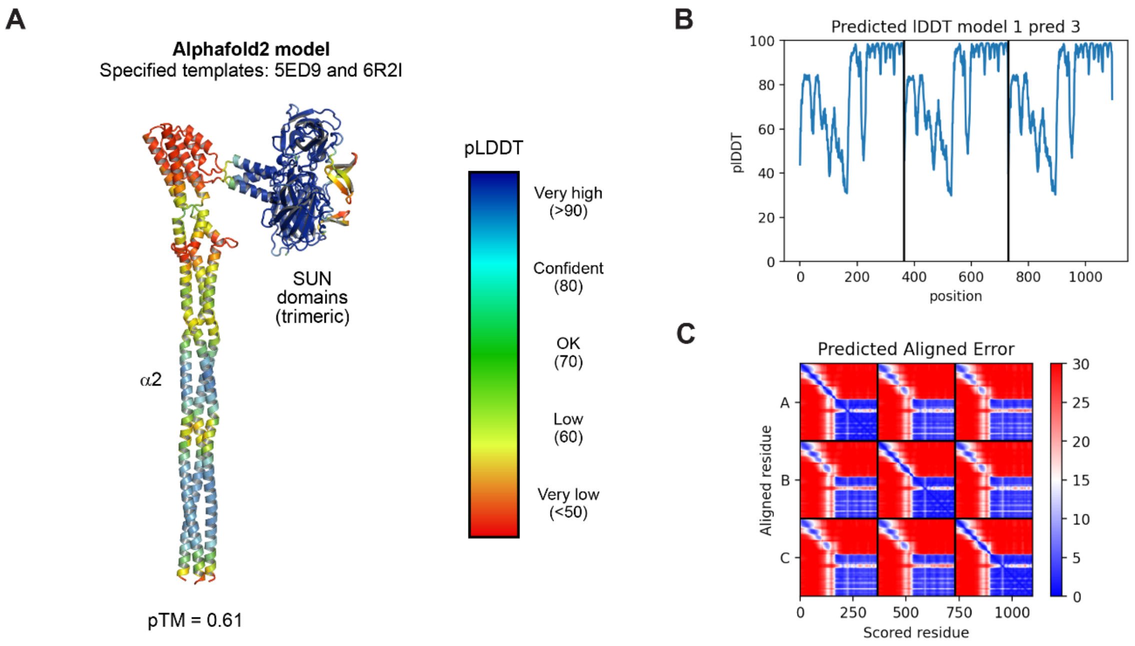

Supplementary Figure S7

Modelling of SUN1 α2-SUN in trimeric SUN domain conformation.

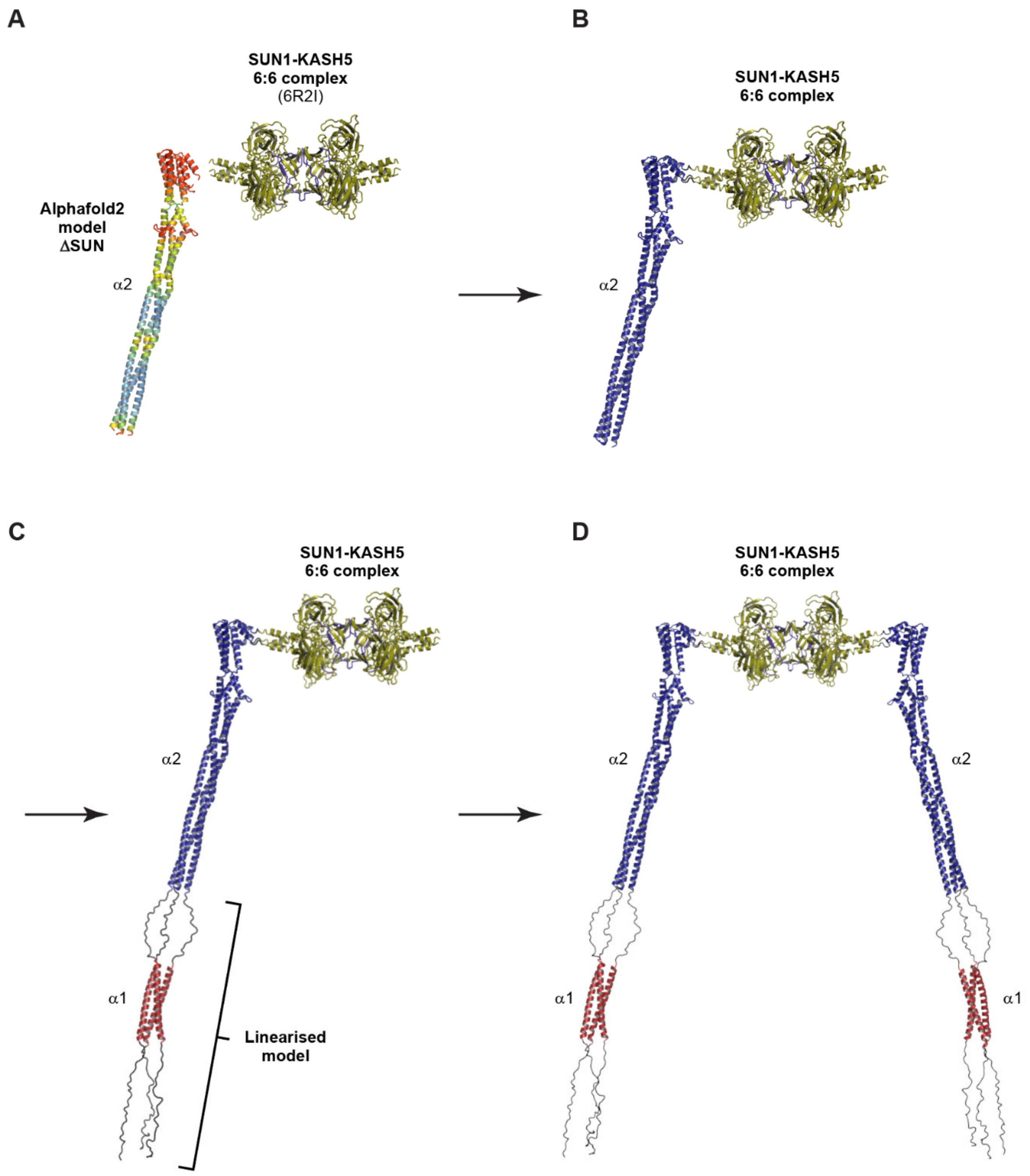

Supplementary Figure S8

Modelling of the SUN1-KASH5 luminal 6:6 complex.

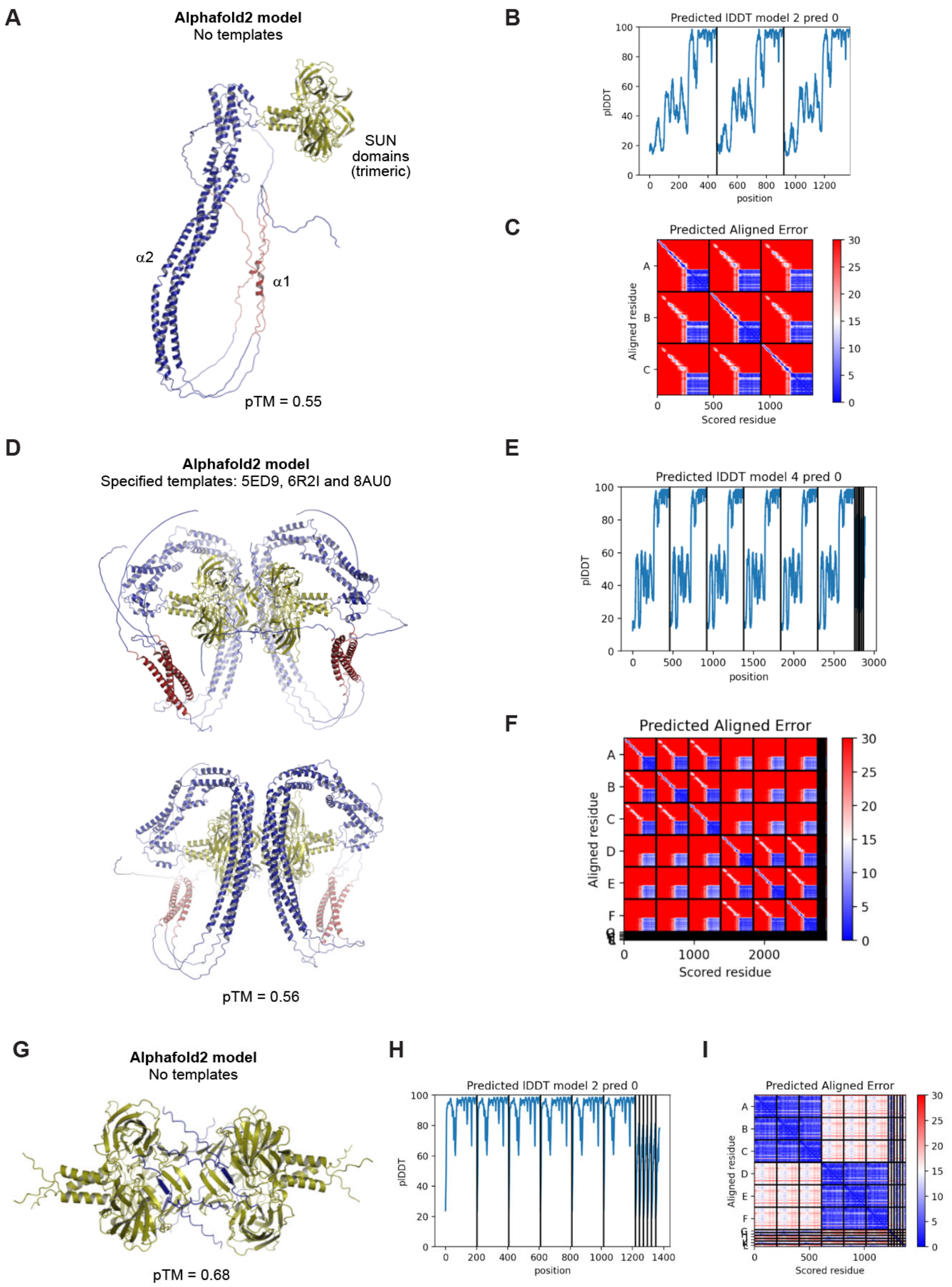

Supplementary Figure S9

Alphafold2 **predictions for comparative purposes.**
