# Supplementary material for: Molecular insights into LINC complex architecture through the crystal structure of a luminal trimeric coiled-coil domain of SUN1
Source: Front Cell Dev Biol. 2023 Jun 21;11:1144277. doi: 10.3389/fcell.2023.1144277 (PMC10320395; doi:10.3389/fcell.2023.1144277)
Supplement: Supplementary file 2 [file DataSheet1.docx]

**Supplementary Figure Legends**

**Supplementary Figure S1**

**SDS-PAGE of protein samples used in this study.**

**Supplementary Figure S2**

**Crystal structure of the SUN1 α1 trimer.**

2Fo-Fc electron density map (1.5σ) of the SUN1 α1 trimer superimposed on its refined crystallographic model.

**Supplementary Figure S3**

**Molecular dynamics simulations of the** **SUN1 α1 trimer.**

2D r.m.s. deviation plots (top) and secondary structure composition per amino-acid with α-helical structure coloured in red (bottom) for the three replicates of 1-μs molecular dynamics simulations of the SUN1 α1 trimeric coiled-coil structure shown in Figure 4.

**Supplementary Figure S4**

**Solution SEC-SAXS analysis of the SUN1 luminal constructs.**

SEC-SAXS analysis of SUN1 (**A,B**) α1, (**C,D**) α2, (**E,F**) α1-α2 and (**G,H**) α1-α2-SUN. (**A,C,E,G**) SAXS Guinier analysis to determine the radius of gyration (*Rg*); linear fits are shown in red, with the fitted data range highlighted in black and demarcated by dashed lines. The *Q*.*Rg* values were < 1.3 and *Rg* was calculated as 20 Å, 51 Å, 70 Å and 69 Å, respectively. (**B,D,F,H**) SAXS Guinier analysis to determine the radius of gyration of the cross-section (*Rc*); linear fits are shown in red, with the fitted data range highlighted in black and demarcated by dashed lines. The *Q*.*Rc* values were < 1.3 and *Rc* was calculated as 10 Å, 13 Å, 14 Å, and both 23 Å and 10 Å, respectively.

**Supplementary Figure S5**

**Circular dichroism (CD) analysis.**

Far UV circular dichroism (CD) spectra recorded between 260 nm and 185 nm in mean residue ellipticity, MRE ([θ]) (x1000 deg.cm^2^.dmol^−1^.residue^−1^).

**Supplementary Figure S6**

**Modelling of the SUN1 luminal trimer.**

(**A**) A model of the SUN1 luminal trimer (amino-acids 326-785) was generated in *Alphafold2* multimer by specifying the use of the SUN1 auto-inhibited SUN domain structure (PDB accession 5YWZ; Xu et al., 2018), SUN2 CC2 that is equivalent to SUN1 α2 (PDB accession 5ED9; Nie et al., 2016), and the SUN1 α1 structure reported herein (PDB accession 8AU0), as the sole structural templates. The resultant model shows the presence of flexible linkers between structured α1 and α2 domains. To represent its structure within biological context, the domains were positioned in line, and intervening linkers were re-modelled into ‘relaxed’ linear conformations (right). Models are coloured according to their pLDDT scores, between blue (>90) and red (<50), as shown. (**B**) Representation of the multiple sequence alignments generated and used by *Alphafold2*, showing the number of sequences and sequence identity against the position along the SUN1 query sequence. (**C**) Predicted LDDT (pLDDT) scores shown for each amino-acid of the three chains of the *Alphafold2*-modelled trimer. Peaks are clearly visible for α1, α2 and SUN domains. (**D**) Predicted aligned error scores between each amino-acid of the three chains of the Alphafold2-modelled trimer, between blue (low error) and red (high error). The α1 and α2 domains show low predicted aligned error both within and between each chain, showing confidence of their trimeric states. The SUN domains show low predicted aligned error within each chain, but not between chains, consistent with their adoption of auto-inhibited rather than trimeric conformations.

**Supplementary Figure S7**

**Modelling of SUN1 α2-SUN in trimeric SUN domain conformation.**

(**A**) A model of the SUN1 α2-SUN luminal trimer (amino-acids 421-785) was generated in *Alphafold2* multimer by specifying the use of the SUN1-KASH5 6:6 complex (PDB accession 6R2I; Gurusaran and Davies, 2021) and SUN2 CC2 that is equivalent to SUN1 α2 (PDB accession 5ED9; Nie et al., 2016) as the sole structural templates. The resultant model shows an approximately 90° hinge between α2 and trimeric SUN domains. The model is coloured according to pLDDT scores, between blue (>90) and red (<50), as shown. The hinge between α2 and trimeric SUN domains has low pLDDT score, indicating low confidence of its predicted conformation, consistent with its inherent flexibility permitting the SUN domain trimer to vary its angulation relative to the α2 domain in respond to tension forces. (**B**) Predicted LDDT (pLDDT) scores shown for each amino-acid of the three chains of the *Alphafold2*-modelled trimer. Peaks are clearly visible for α2 and SUN domains. (**C**) Predicted aligned error scores between each amino-acid of the three chains of the *Alphafold2*-modelled trimer, between blue (low error) and red (high error). The α2 domains show low predicted aligned error both within and between each chain, showing confidence of its trimeric state. The SUN domains show similarly low predicted aligned error show low predicted aligned error both within and between each chain (in contrast to the auto-inhibited SUN1 luminal trimer), consistent with their adoption of a KASH-binding trimeric rather that auto-inhibited conformations.

**Supplementary Figure S8**

**Modelling of the SUN1-KASH5 luminal 6:6 complex.**

(**A**) The trimeric SUN domain of the Alphafold2 multimer model of α2-SUN (Supplementary Figure 6) was deleted, and the remaining structure was positioned adjacent to the SUN1-KASH6 6:6 core structure (PDB accession 6R2I). (**B**) The α2 domain and a KASH5-bound SUN trimer of the SUN1-KASH5 structure were joined by subtle re-remodelling of the flexible linker. (**C**) The resultant structure was then joined to the α1 domain and surrounding flexible linkers from the SUN1 luminal trimer model (Supplementary Figure 6). (**D**) The full 6:6 luminal complex was completed by superimposing a copy of the full 3:3 structure from one side of the complex onto the remaining KASH5-bound SUN trimer of the SUN1-KASH5 structure.

**Supplementary Figure S9**

***Alphafold2* predictions for comparative purposes.**

(**A**) A model of the SUN1 luminal trimer (amino-acids 326-785) was generated in *Alphafold2* multimer using no templates. (**B**) Predicted LDDT (pLDDT) scores shown for each amino-acid of the three chains of the *Alphafold2*-modelled trimer. (**C**) Predicted aligned error scores between each amino-acid of the three chains of the *Alphafold2*-modelled trimer, between blue (low error) and red (high error). (**D**) A model of the SUN1-KASH5 luminal 6:6 complex was generated in *Alphafold2* multimer by specifying the use of the SUN1-KASH5 core structure (PDB accession 6R2I; Gurusaran and Davies, 2021), SUN2 CC2 that is equivalent to SUN1 α2 (PDB accession 5ED9; Nie et al., 2016), and the SUN1 α1 structure reported herein (PDB accession 8AU0), as the sole structural templates. This has the same topology as the model reported herein. (**E**) Predicted LDDT (pLDDT) scores shown for each amino-acid of the six SUN1 and six KASH5 chains of the *Alphafold2*-modelled complex. (**F**) Predicted aligned error scores between each amino-acid of the six SUN1 and six KASH5 chains of the *Alphafold2*-modelled complex, between blue (low error) and red (high error). (**G**) A model of the SUN1-KASH5 core 6:6 complex was generated in *Alphafold2* multimer using no templates. (**H**) Predicted LDDT (pLDDT) scores shown for each amino-acid of the six SUN1 and six KASH5 chains of the *Alphafold2*-modelled complex. (**I**) Predicted aligned error scores between each amino-acid of the six SUN1 and six KASH5 chains of the *Alphafold2*-modelled complex, between blue (low error) and red (high error).
